# Reappraisal of the phylogenetic relationships of hirsutella-like fungi across *Ophiocordycipitaceae* and *Polycephalomycetaceae* (*Hypocreales*, *Sordariomycetes*), and the description of seven new species

**DOI:** 10.3897/imafungus.17.171084

**Published:** 2026-02-17

**Authors:** Xian Zhang, Xing-Can Peng, De-Ping Wei, Yi Wang, Chada Norphanphoun, Sinang Hongsanan, Ning Xie, Zhong-Liang Liu, Ausana Mapook, Ji-Chuan Kang, Natsaran Saichana, Kevin D. Hyde, Ting-Chi Wen

**Affiliations:** 1 State Key Laboratory of Green Pesticide, Key Laboratory of Green Pesticide and Agricultural Bioengineering, Ministry of Education, Guizhou University, Guiyang 550025, China Center of Excellence in Fungal Research, Mae Fah Luang University Chiang Rai Thailand; 2 Engineering Research Center of Southwest Bio-Pharmaceutical Resources, Ministry of Education, Guizhou University, Guiyang 550025, China School of Science, Mae Fah Luang University Chiang Rai Thailand; 3 Center of Excellence in Fungal Research, Mae Fah Luang University, Chiang Rai 57100, Thailand College of Life Science and Oceanography, Shenzhen University Shenzhen China; 4 School of Science, Mae Fah Luang University, Chiang Rai 57100, Thailand Engineering Research Center of Southwest Bio-Pharmaceutical Resources, Ministry of Education, Guizhou University Guiyang China; 5 Department of Plant Pathology, Agricultural College, Guizhou University, Guiyang, 550025, China Agricultural College, Guizhou University Guiyang China; 6 Shenzhen Key Laboratory of Microbial Genetic Engineering, College of Life Science and Oceanography, Shenzhen University, Shenzhen 518060, China State Key Laboratory of Green Pesticide, Key Laboratory of Green Pesticide and Agricultural Bioengineering, Ministry of Education, Guizhou University Guiyang China

**Keywords:** Entomopathogenic fungi, hirsutella-like, new taxa, pairwise homoplasy index, phylogeny, taxonomy

## Abstract

Entomopathogens constitute a unique and specialized trophic group of fungi, most of which belong to *Hypocreales* (*Sordariomycetes*, *Ascomycota*). In this study, eight species were collected and isolated from China and Thailand. Through comprehensive morphological analyses and multigene phylogenetic studies (ITS, nrSSU, nrLSU, *tef*1-α, *rpb*1, *rpb*2), seven novel species (*Ophiocordyceps jinguangensis***sp. nov**., *O. northeastensis***sp. nov**., *Polycephalomyces bannaensis***sp. nov**., *Po. chiangraiensis***sp. nov**., *Pleurocordyceps shibingensis***sp. nov**., *Pl. tengchongensis***sp. nov**., and *Dingleyomyces yunnanensis***sp. nov**.) and one known species (*O. formicarum*) were identified. Additionally, the pairwise homoplasy index (PHI) test results and morphological differences between the new species and their closely related taxa are provided. Notably, as the number of reported hirsutella-like species continues to increase, their phylogenetic placement has become increasingly unclear in previous classifications. To address this issue, this paper presents the first comprehensive summary of the distribution of hirsutella-like species within the families *Ophiocordycipitaceae* and *Polycephalomycetaceae*, along with an analysis of the similarities and differences in their phialidic characteristics. These findings significantly expand our knowledge of the diversity, taxonomy, and phylogenetic relationships of entomopathogenic fungi in these families, providing a valuable framework for future studies on their ecology and evolution.

## Introduction

Entomopathogenic fungi represent a group specialized in infecting insects. These organisms are distributed across five major fungal phyla, with the highest taxonomic diversity concentrated within *Hypocreales* (Araújo and Hughes 2016; Thiyagaraja et al. 2025). These fungi are classified into four families: *Clavicipitaceae*, *Cordycipitaceae*, *Ophiocordycipitaceae*, and *Polycephalomycetaceae* (Sung et al. 2007; Xiao et al. 2023). However, species possessing hirsutella-like asexual morphs are primarily distributed in the families *Ophiocordycipitaceae* and *Polycephalomycetaceae*. *Ophiocordycipitaceae* was proposed by Sung et al. (2007) and originally classified as a subgenus within *Cordyceps*. Over time, *Ophiocordycipitaceae* has undergone extensive taxonomic revisions integrating morphological and phylogenetic analyses to resolve its classification and delineate boundaries among constituent genera (Sung et al. 2007; Quandt et al. 2014; Mongkolsamrit et al. 2020; Crous et al. 2020; Araújo et al. 2022; Xiao et al. 2023). Notably, *Ophiocordyceps*, the type genus of this family, is recognized for its dark, fibrous to wiry stromata and perithecia, with a wide range of hosts (Sung et al. 2007; Ban et al. 2015; Araújo et al. 2018; Xiao et al. 2019). Other genera within *Ophiocordycipitaceae* also display remarkable morphological diversity and ecological strategies. *Drechmeria* includes soilborne nematophagous fungi characterized by solitary or verticillate conidiophores (Yu et al. 2018). *Harposporium* comprises obligate endoparasites of nematodes and rotifers, producing distinctive sickle-shaped conidia (Wang et al. 2007; Yadav et al. 2023). *Tolypocladium* is notable for its clavicipitoid sexual morphs and production of bioactive secondary metabolites, including cyclosporin derivatives (Yu et al. 2021). *Purpureocillium*, best represented by *P. lilacinum*, includes opportunistic entomopathogenic and nematophagous species with characteristic lilac to purple conidiophores (Luangsa-ard et al. 2011). Mongkolsamrit et al. (2019) resurrected the genus *Paraisaria* by introducing three new species. Recently established genera *Hantamomyces* and *Torrubiellomyces* further broaden the morphological and ecological range of the family (Crous et al. 2020; Araújo et al. 2022). Together, these genera illustrate the broad evolutionary diversification and ecological adaptability of *Ophiocordycipitaceae*. *Polycephalomycetaceae*, on the other hand, exhibits remarkable ecological adaptability, parasitizing diverse insect and fungal hosts across tropical and subtropical regions. This family was established by Xiao et al. (2023) to accommodate *Perennicordyceps*, *Pleurocordyceps*, and *Polycephalomyces*, which were previously assigned to *Ophiocordycipitaceae*. *Polycephalomycetaceae* is supported as a distinct lineage based on combined morphological characters from both sexual and asexual morphs, together with multigene phylogenetic analyses (ITS, nrSSU, nrLSU, *tef*1-α, *rpb*1, *rpb*2). *Pleurocordyceps* is characterized by stipitate synnemata, acremonium- to hirsutella-like phialides, and dimorphic conidia, representing a well-supported lineage in *Polycephalomycetaceae*. *Polycephalomyces* is a morphologically conservative but phylogenetically distinct genus, mainly parasitizing insects and other fungi, and is characterized by synnematous asexual morphs with hirsutella-like phialides. *Dingleyomyces*, a parasitic genus on the stromata of large *Ophiocordyceps* species, was introduced by Johnston et al. (2023). The genus was previously placed in *Torrubiella* as *T. lloydii*. Wang et al. (2024) established *Paradingleyomyces* to accommodate the monotypic species *Pa. lepidopterorum* based on morphological characteristics and multigene phylogenetic analysis.

Hirsutella-like fungi display substantial morphological plasticity, often obscuring generic and species boundaries. As a result, hirsutella-like fungi have played a central role in debates over taxonomic delimitation and evolutionary relationships within these families, warranting focused discussion. Speare (1920) originally defined *Hirsutella* as synnematous fungi with basally inflated phialides arranged in a hymenial layer and mucous-sheathed conidia. Subsequent studies expanded the genus to include polyphialides, non-inflated phialides, capitate synnemata, non-mucous conidia, didymoconidia, and multiple synanamorphs, reflecting greater morphological diversity (Hodge 1998). Quandt et al. (2014) noted that hirsutella-like fungi are widely distributed within *Ophiocordyceps*. The resulting morphological ambiguity, ecological overlap, and the need for taxonomic stability prompted the transfer of most hirsutella-like taxa into *Ophiocordyceps*. Consequently, newly discovered hirsutella-like fungi have predominantly been classified under *Ophiocordyceps*. Simmons et al. (2015) established a foundational classification system for hirsutella-like anamorphs, delineating six subclades: *H. citriformis*, *H. guyana*, *H. nodulosa*, *H. sinensis*, *H. thompsonii*, and a distinct “ant pathogen” subclade. Qu et al. (2018b) later provided comprehensive morphological descriptions for the first five subclades, while Araújo et al. (2018) redefined the “ant pathogen” group as the *O. unilateralis* clade. Subsequent studies expanded the phylogenetic framework, proposing additional hirsutella-like clades such as *O. sobolifera* and *O. ravenelii* (Wang et al. 2018; Sun et al. 2024). For instance, Dai et al. (2024) merged four subclades (*H. guyana*, *H. nodulosa*, *H. sinensis*, and *H. thompsonii*) into a broader *O. sinensis* clade, while Xie et al. (2025) proposed two novel clades (*O. issidarum* and *O. acicularis*), which have not previously been recognized. These taxonomic inconsistencies highlight the necessity of synthesizing and stabilizing the phylogenetic framework of clades characterized by hirsutella-like anamorphs.

In this study, we collected eight entomopathogenic fungi from Guizhou, Yunnan, and Liaoning Provinces in China, as well as Chiang Rai Province in Thailand. Three of the eight species were recognized as members of *Ophiocordyceps*, and five species were classified within *Polycephalomycetaceae*, based on macroscopic and microscopic characteristics. A phylogenetic analysis using ITS, nrSSU, nrLSU, *tef*1-α, *rpb*1, and *rpb*2 was conducted to clarify the taxonomic placements of these species. In addition, given the long-standing taxonomic complexity of hirsutella-like fungi, we further summarized the distribution and morphological diversity of hirsutella-like clades across *Ophiocordycipitaceae* and *Polycephalomycetaceae*. This overview provides essential phylogenetic context for interpreting the newly described species and establishes a clearer framework for evaluating convergent phialide morphologies within these families.

## Materials and methods

### Sample collection, morphological observation, and isolation

Insect specimens infected with entomopathogenic fungi were collected from Guizhou, Liaoning, and Yunnan Provinces in China, as well as from Chiang Rai Province in Thailand. Specimens were obtained from the lower and upper surfaces of living leaves and from leaf litter in both evergreen and deciduous forests with low sunlight exposure. The hosts of some specimens were buried in soil, such as *Polycephalomyces bannaensis*. Fresh specimens were photographed in the field using a mobile phone camera (Huawei P40), placed in plastic boxes, and transported to the laboratory for further analysis. Fresh fruiting bodies were examined using a stereomicroscope (Leica S9E). Freehand sections of fertile structures were prepared, placed on glass slides, and mounted in either ultrapure water or lactophenol cotton blue solution for microscopic observation using a Leica DM2500 compound microscope equipped with a digital camera and measured using Leica imaging software. The photographic plates were prepared using Adobe Photoshop CC (2018). Fungal isolation was initiated on the day of collection to preclude contamination. A small mass of conidia from synnemata or sclerotia within insect hosts was transferred to potato dextrose agar (PDA) plates using a sterile needle (Wei et al. 2021). Cultures were incubated at 25 °C in the dark for 2 weeks until colonies reached 2–3 cm in diameter. Herbarium specimens were deposited in the Kunming Institute of Botany, Academia Sinica (HKAS), and Mae Fah Luang University (MFLU). Living cultures grown on PDA were deposited in the Kunming Institute of Botany Culture Collection (KUNCC) and the Mae Fah Luang University Culture Collection (MFLUCC). Index Fungorum (IF) numbers were registered following the protocol outlined by Jayasiri et al. (2015) and Index Fungorum (www.indexfungorum.org, accessed on 3 September 2025).

### DNA extraction, amplification, and sequencing

Total genomic DNA was extracted from fresh mycelia grown on PDA medium and fungal tissues from dry specimens using a DNA extraction kit (Omega Fungus Genomic DNA Extraction Kit, China), following the manufacturer’s instructions. This study used ITS, nrSSU, nrLSU, *tef*1-α, *rpb*1, and *rpb*2. The ITS region was amplified with primers ITS5 and ITS4, while nrSSU was amplified using NS1 and NS4 (White et al. 1990). The nrLSU region was amplified with the primer pair LR0R and LR5 (Vilgalys and Hester 1990). The *tef*1-α gene was amplified with primers EF1-983F and EF1-2218R (Rehner and Buckley 2005). The *rpb*1 region was targeted using primers CRPB1A and RPB1Cr (Castlebury et al. 2004), while *rpb*2 was amplified with primers fRPB2-5f and fRPB2-7cR (Liu et al. 1999). The PCR amplification conditions for ITS, nrLSU, nrSSU, *tef*1-α, *rpb*1, and *rpb*2 followed a protocol consisting of (1) an initial denaturation at 94 °C for 3 min; (2) 33 cycles of denaturation at 94 °C for 30 s, followed by locus-specific annealing (ITS: 51 °C for 50 s; nrSSU: 47 °C for 1 min 20 s; nrLSU: 50 °C for 30 s; *tef*1-α: 58 °C for 50 s; *rpb*1 and *rpb*2: 51 °C for 40 s) and extension (ITS: 72 °C for 45 s; nrSSU and nrLSU: 72 °C for 1 min 50 s; *tef*1-α: 72 °C for 1 min; *rpb*1 and *rpb*2: 72 °C for 1 min 20 s); and (3) a final extension at 72 °C for 10 min. All PCR products were sequenced at Tsingke Biological Technology in Chongqing, China. The newly generated sequences in this study were deposited in GenBank (www.ncbi.nlm.nih.gov/genbank/) and used for the phylogenetic analysis. Information on all sequences used for the phylogenetic analysis is provided in Table 1.

**Table 1.** GenBank accession numbers of the taxa used in the phylogenetic analyses; newly generated sequences are shown in bold.

Species	Voucher number	GenBank accession number						Reference
		ITS	nrSSU	nrLSU	*tef1-α*	*rpb1*	*rpb2*	
*Cordyceps pleuricapitata*	NBRC 100745	JN943304	JN941750	JN941391	KF049679	JN992484	KF049667	Kepler et al. (2013)
*Cordyceps pleuricapitata*	NBRC 100746	JN943306	JN941749	JN941392	KF049680	JN992483	KF049668	Kepler et al. (2013)
*Dingleyomyces lloydii*	PDD 121254	OR602634	OR647563	OR602640	OR588853	OR588860	OR588858	Johnston et al. (2023)
***Dingleyomyces yunnanensis***	**HKAS 149970**		**PX692988**	**PX688518**	**PX694739**	**PX694719**		**This study**
***Dingleyomyces yunnanensis***	**HKAS 149969**		**PX692989**	**PX688519**	**PX694740**	**PX694720**		**This study**
*Drechmeria balanoides*	CBS 250.82	MH861495	AF339588	AF339539	DQ522342	DQ522388	DQ522442	Sung et al. (2007)
*Drechmeria campanulata*	IMI 356051	NR_155045	NG_064867	NG_059063	–	–	–	Zare et al. (2000)
*Drechmeria coniospora*	CBS 596.92	AF106018	AF106012	–	–	–	–	Gernandt et al. (1999)
*Drechmeria glocklingiae*	CBS 101434	AJ292418	–	–	–	–	–	Zare et al. (2000)
*Drechmeria gunnii*	OSC 76404	JN049822	AF339572	AF339522	AY489616	AY489650	DQ522426	Luangsa-ard et al. (2018)
*Drechmeria panacis*	CBS 142798	MF588878	MF588890	MF588897	MF614144	–	–	Yu et al. (2018)
*Drechmeria rhabdospora*	CBS 101432	AF375050	–	–	–	–	–	Zare et al. (2001)
*Drechmeria sinensis*	CBS 567.95	MH862540	AF339594	AF339545	DQ522343	DQ522389	DQ522443	Spatafora et al. (2007)
*Drechmeria sphaerospora*	CBS 522.80	–	AF339590	AF339541	–	–	–	Sung et al. (2001)
*Drechmeria zeospora*	CBS 335.80	MH861269	AF339589	AF339540	EF469062	EF469091	EF469109	Sung et al. (2007)
*Hantamomyces aloidendri*	CPC 38655	MW175348	–	MW175388	–	–	–	Crous et al. (2020)
*Harposporium anguillulae*	ARSEF 5407	–	–	AY636080	–	–	–	Chaverri et al. (2005)
*Harposporium bysmatosporum*	BCRC 34226	FJ380935	–	–	–	–	–	Chen et al. (2024)
*Harposporium cerberi*	CBS 129580	MH865377	–	–	–	–	–	Vu et al. (2019)
*Harposporium cycloides*	ARSEF 5599	–	–	AY636083	–	–	–	Chaverri et al. (2005)
*Harposporium harposporiferum*	ARSEF 5472	–	AF339569	NG_060621	–	–	–	Sung et al. (2001)
*Harposporium helicoides*	Arsef 5354	–	AF339577	AF339527	–	–	–	Sung et al. (2001)
*Harposporium illinoisense*	CPC 42872	OQ990112	–	OQ990063	OQ989244	–	OQ989214	Crous et al. (2023)
*Harposporium incensis*	ZBAC1472	OQ161195	–	–	OQ186688	OQ186690	OQ186692	Chen et al. (2025)
*Harposporium peltatum*	ARSEF 5410	–	–	AY636082	–	–	–	Chaverri et al. (2005)
*Hirsutella changbeisanensis*	GZUlFR hirl60527	KY415578	–	–	KY415592	–	–	Qu et al. (2017)
*Hirsutella citriformis*	ARSEF 490	KM652151	–	KM652103	KM651987	–	–	Simmons et al. (2015)
*Hirsutella cryptosclerotium*	ARSEF 4517	KM652157	KM652066	KM652109	KM651992	KM652032	–	Simmons et al. (2015)
*Hirsutella fusiformis*	ARSEF 5474	–	KM652067	KM652110	KM651993	KM652033	–	Simmons et al. (2015)
*Hirsutella gigantea*	ARSEF 30	–	–	JX566977	JX566980	KM652034	–	Simmons et al. (2015)
*Hirsutella guyana*	ARSEF 878	KM652158	KM652068	KM652111	KM651994	KM652035	–	Simmons et al. (2015)
*Hirsutella haptospora*	ARSEF 2226	KM652159	–	–	KM651995	KM652036	–	Simmons et al. (2015)
*Hirsutella heteroderae*	CBS:216.78	–	–	MH872887	–	–	–	Vu et al. (2019)
*Hirsutella homalodiscae*		–	–	DQ075674	–	–	–	Boucias et al. (2007)
*Hirsutella huangshanensis*	RCEF0868	EF689043	–	–	–	–	–	Pérez-González et al. (2015)
*Hirsutella illustris*	ARSEF 5539	KM652160	KM652069	AY518380	KM651996	KM652037	–	Simmons et al. (2015)
*Hirsutella kirchneri*	ARSEF 5551	KM652161	KM652070	KM652113	KM651997	–	–	Simmons et al. (2015)
*Hirsutella kuankuoshuiensis*	GZUIFR-2012KKS3-1	KY415575	–	KY415582	KY415590	KY945360	–	Qu et al. (2021)
*Hirsutella lecaniicola*	ARSEF 8888	KM652162	KM652071	KM652114	KM651998	KM652038	–	Simmons et al. (2015)
*Hirsutella leizhouensis*	GZUIFR-hir130707	KY415573	–	KY415580	KY415587	KY945358	–	Qu et al. (2017)
*Hirsutella liboensis*	ARSEF 9603	KM652163	KM652072	KM652115	–	–	–	Simmons et al. (2015)
*Hirsutella necatrix*	ARSEF 5549	KM652164	KM652073	KM652116	KM651999	KM652039	–	Simmons et al. (2015)
*Hirsutella nodulosa*	ARSEF 5473	KM652165	KM652074	KM652117	KM652000	KM652040	–	Simmons et al. (2015)
*Hirsutella radiata*	ARSEF 1369	–	KM652076	KM652119	KM652002	KM652042	–	Simmons et al. (2015)
*Hirsutella rhossiliensis*	ARSEF 2931	KM652168	KM652078	KM652121	KM652004	KM652043	–	Simmons et al. (2015)
*Hirsutella satumaensis*	ARSEF 996	KM652172	KM652082	KM652125	KM652008	KM652047	–	Simmons et al. (2015)
*Hirsutella shennongjiaensis*	GZUIFR-Snj121022	KT390721	–	KY945357	–	KY945364	–	Zou et al. (2016)
*Hirsutella sinensis*	HMAS 55469	AJ243980	–	–	–	–	–	Zhao et al. (1999)
*Hirsutella stilbelliformis*	Q2	–	EU864318	–	–	–	–	Hughes et al. (2009)
*Hirsutella strigosa*	ARSEF 2197	KM652175	KM652085	KM652129	KM652012	KM652050	–	Simmons et al. (2015)
*Hirsutella subulata*	ARSEF 2227	KM652176	KM652086	KM652130	KM652013	KM652051	–	Simmons et al. (2015)
*Hirsutella thompsonii*	ARSEF 241	KM652178	–	KM652132	KM652015	–	–	Simmons et al. (2015)
*Hirsutella versicolor*	ARSEF 1037	–	KM652102	KM652150	KM652029	KM652063	–	Simmons et al. (2015)
*Ophiocordyceps acicularis*	OSC128580	JN049820	DQ522543	DQ518757	DQ522326	DQ522371	–	Sung et al. (2007)
*Ophiocordyceps acroasca*	YFCC 9016	–	ON555841	ON555922	ON567761	ON568681	ON568134	Tang et al. (2023)
*Ophiocordyceps agriotidis*	ARSEF 5692	JN049819	DQ522540	DQ518754	DQ522322	DQ522368	DQ522418	Kepler et al. (2012)
*Ophiocordyceps albacongiuae*	RC20	–	KX713633	–	KX713670	–	–	Araújo et al. (2018)
*Ophiocordyceps appendiculata*	NBRC 106960	JN943326	JN941728	JN941413	AB968577	JN992462	AB968539	Ban et al. (2015)
*Ophiocordyceps appendiculata*	NBRC 106959	JN943325	JN941729	JN941412	AB968578	JN992463	AB968540	Ban et al. (2015)
*Ophiocordyceps arborescens*	NBRC 105891	AB968398	AB968386	AB968414	AB968572	–	AB968534	Ban et al. (2015)
*Ophiocordyceps asiatica*	BCC 30516	MH754722	–	MH753675	MK284263	MK214105	MK214091	Tasanathai et al. (2019)
*Ophiocordyceps australis*	HUA 186097	KF937350	KC610786	KC610765	KC610735	KF658662	–	Sanjuan et al. (2015)
*Ophiocordyceps barnesii*	BCC 28560	–	EU408776	–	–	EU408773	EU418599	Sanjuan et al. (2015)
*Ophiocordyceps basiasca*	YHH 20191	–	ON555828	ON555910	ON567748	ON568672	ON568121	Tang et al. (2023)
*Ophiocordyceps bidoupensis*	YHH 20036	–	OK571396	–	OK556893	OK556897	OK556899	Zou et al. (2022)
*Ophiocordyceps bifertilis*	YFCC 9012	–	ON555843	ON555923	ON567763	ON568143	ON568135	Tang et al. (2023)
*Ophiocordyceps bispora*	KVL 606	–	AH006986	AF009654	–	–	–	Suh et al. (1998)
*Ophiocordyceps blakebarnesii*	MISSOU3	–	KX713643	KX713608	KX713687	KX713714	–	Araújo et al. (2018)
*Ophiocordyceps borealis*	MFLU 18-0163	MK863251	MK863044	MK863051	MK860189	–	–	Zha et al. (2021)
*Ophiocordyceps brunnea*	BBH 49819	–	–	–	OR855788	OR855808	OR855832	Mongkolsamrit et al. (2024)
*Ophiocordyceps brunneinigra*	BCC 69015	–	–	MF614653	MF614637	–	MF614680	Luangsa-ard et al. (2018)
*Ophiocordyceps brunneiperitheciata*	BCC 49312	–	–	MF614660	MF614642	–	MF614686	Luangsa-ard et al. (2018)
*Ophiocordyceps brunneipunctata*	OSC 128576	–	DQ522542	DQ518756	DQ522324	DQ522369	DQ522420	Spatafora et al. (2007)
*Ophiocordyceps campes*	BCC36938	MT783955	–	MT118175	MT118167	MT118183	MT118188	Tasanathai et al. (2020)
*Ophiocordyceps camponoti-atricipis*	ATRI3	–	KX713666	KX520652	KX713677	–	–	Araújo et al. (2018)
*Ophiocordyceps camponoti-femorati*	FEMO2	–	KX713663	KX713590	KX713678	KX713702	–	Araújo et al. (2018)
*Ophiocordyceps camponoti-novogranadensis*	Mal63	–	KX713648	KX713603	–	–	–	Araújo et al. (2018)
*Ophiocordyceps capilliformis*	BCC 82180	–	–	–	OR855794	OR855814	OR855837	Mongkolsamrit et al. (2024)
*Ophiocordyceps citrina*	TNS F18537	–	–	KJ878903	KJ878983	–	–	Quandt et al. (2014)
*Ophiocordyceps clavata*	NBRC 106961	JN943327	JN941727	JN941414	AB968586	JN992461	AB968547	Schoch et al. (2012)
*Ophiocordyceps coccidiicola*	NBRC 100682	AB968404	AB968391	AB968419	AB968583	–	AB968545	Ban et al. (2015)
*Ophiocordyceps communis*	BCC 1842	MH754726	–	MH753680	MK284266	MK214110	MK214096	Tasanathai et al. (2019)
*Ophiocordyceps contiispora*	YFCC 9027	–	ON555832	ON555913	ON567752	ON568142	ON568125	Tang et al. (2023)
*Ophiocordyceps corriemoreauae*	C2014A	–	MK393831	MK393324	–	MK491926	–	Araújo et al. (2020)
*Ophiocordyceps cystidiata*	GZUIFR-2023XY-OA5C	–	PQ497594	PQ497634	–	PQ516632	PQ516636	Xu et al. (2025)
*Ophiocordyceps daceti*	MF01	–	–	KX713604	KX713667	–	–	Araújo et al. (2018)
*Ophiocordyceps delicatula*	ARSEF 14442	–	MZ198251	–	MZ246828	MZ246829	–	Clifton et al. (2021)
*Ophiocordyceps desmidiospora*	SJS3Des	–	MH536515	MH536514	MN785129	MN785131	–	Saltamachia et al. (2020)
*Ophiocordyceps elongata*	OSC 110989	–	–	EF468808	EF468748	EF468856	–	Sung et al. (2007)
*Ophiocordyceps emeiensis*	G96031	AJ309347	–	–	–	–	–	Liu et al. (2002)
*Ophiocordyceps entomorrhiza*	ARSEF:13375	–	MH057734	–	MH057732	MH057733	–	Wraight et al. (2018)
*Ophiocordyceps fenggangensis*	HKAS 125848T	OR527535	–	OR527542	OR526346	OR526351	–	Peng et al. (2024)
*Ophiocordyceps flabellata*	YFCC 8795	–	OL310721	OL310724	OL322688	OL322687	OL322695	Tang et al. (2023)
*Ophiocordyceps flavida*	BCC 84256	–	–	MT512655	MT533482	MT533476	–	Mongkolsamrit et al. (2021)
*Ophiocordyceps formicarum*	BCMU CF01	AB222678	–	–	–	–	–	Freire (2015)
*Ophiocordyceps formicarum*	TNS F18565	–	KJ878921	KJ878888	KJ878968	KJ879002	KJ878946	Quandt et al. (2014)
***Ophiocordyceps formicarum***	HKAS 149980	**PX692974**	**PX692987**	**PX688517**	**PX694738**			**This study**
*Ophiocordyceps formosana*	MFLU 15 3888	–	KU854951	–	KU854949	KU854947	–	Li et al. (2016)
*Ophiocordyceps formosana*	TNM F13893	–	KJ878908	–	KJ878956	KJ878988	KJ878943	Quandt et al. (2014)
*Ophiocordyceps fulgoromorphila*	HUA 186139	–	KC610794	KC610760	KC610729	KF658676	KC610719	Sanjuan et al. (2015)
*Ophiocordyceps furcatosubulata*	YHH 17005	–	MT774217	MT774224	MT774245	MT774231	MT774238	Wang et al. (2021)
*Ophiocordyceps fusiformis*	BCC 93025	MZ676743	–	MZ675422	MZ707849	MZ707855	MZ707805	Tasanathai et al. (2022)
*Ophiocordyceps geometridicola*	TBRC 8094	–	–	MF614647	MF614631	MF614664	MF614678	Luangsa-ard et al. (2018)
*Ophiocordyceps globiceps*	MFLU 18-0661	MH725816	MH725812	MH725830	MH727388	–	–	Xiao et al. (2019)
*Ophiocordyceps globosa*	BCC 93023	MZ676740	–	MZ675419	MZ707846	MZ707861	–	Tasanathai et al. (2022)
*Ophiocordyceps gracillima*	HUA 186132	KF937353	–	KC610768	KC610744	KF658666	–	Sanjuan et al. (2015)
*Ophiocordyceps granospora*	BCC 82255	MH028143	–	MH028156	MH028183	MH028168	MH028177	Khonsanit et al. (2019)
*Ophiocordyceps halabalaensis*	MY1308	GU723758	–	–	GU797109	–	–	Luangsa-ard et al. (2011)
*Ophiocordyceps hauturu*	PDD:108384	MW191773	–	OR602639	–	–	–	Johnston et al. (2023)
*Ophiocordyceps highlandensis*	HKAS83206-1	–	KM581282	–	–	KM581274	KM581278	Yang et al. (2015)
*Ophiocordyceps hydrangea*	YFCC 8834	–	OM304635	OM304639	OM831276	OM831279	OM831282	Zou et al. (2022)
*Ophiocordyceps indica*	CAL 1880	KX679571	MZ571406	KY486751	MZ514914	MZ514912	–	Shaema et al. (2023)
*Ophiocordyceps irangiensis*	NBRC 101399	JN943334	JN941716	JN941425	–	JN992450	–	Schoch et al. (2012)
*Ophiocordyceps isopterorum*	BCC 93042	MZ676742	–	MZ675421	MZ707848	–	MZ707804	Tasanathai et al. (2022)
*Ophiocordyceps issidarum*	MFLU 17 0751	MF398185	–	MF398188	–	–	–	Hyde et al. (2017)
***Ophiocordyceps jinguangensis***	**HKAS 149981**	**PX692972**	**PX692985**		**PX694736**	**PX694717**	**PX694727**	**This study**
***Ophiocordyceps jinguangensis***	**HKAS 149982**	**PX692973**	**PX692986**		**PX694737**	**PX694718**	**PX694728**	**This study**
*Ophiocordyceps karstii*	MFLU 15 3884	–	KU854952	–	KU854945	KU854943	–	Li et al. (2016)
*Ophiocordyceps khokpasiensis*	BCC 48071	MH754728	–	MH753682	MK284269	MK214112	–	Tasanathai et al. (2019)
*Ophiocordyceps khonkaenensis*	BCC81462	–	MK632126	–	MK632075	MK632168	MK632157	Crous et al. (2019)
*Ophiocordyceps kimflemingiae*	SC100	–	–	KX713624	KX713696	KX713725	–	Araújo et al. (2018)
*Ophiocordyceps kniphofioides*	Ophkni975	–	KC610790	KF658679	KC610739	KF658667	KC610717	Araújo et al. (2018)
*Ophiocordyceps kobayasii*	BCC75694	–	MK632112	MK632082	MK632056	MK632172	MK632136	Thanakitpipattana et al. (2020)
*Ophiocordyceps kohchangensis*	BCC 88229	–	–	–	–	OR855817	–	Mongkolsamrit et al. (2024)
*Ophiocordyceps konnoana*	EFCC 7315	–	EF468959	–	EF468753	EF468861	EF468916	Sung et al. (2007)
*Ophiocordyceps krachonicola*	BCC79666	–	–	MK632080	MK632054	MK632161	MK632132	Thanakitpipattana et al. (2020)
*Ophiocordyceps kuchinaraiensis*	BCC 95830	OQ627396	–	OQ627397	OQ625474	–	OQ625475	Crous et al. (2023)
*Ophiocordyceps lanpingensis*	YHOS0705	–	KC417458	KC417460	KC417462	KC417464	KC456333	Chen et al. (2013)
*Ophiocordyceps laotii*	BCC 76495	ON763786	–	ON764219	ON759347	ON759354	–	Mongkolsamrit et al. (2023)
*Ophiocordyceps liangii*	HKAS 125845T	OR527536	OR527539	OR527543	OR526347	–	–	Peng et al. (2024)
*Ophiocordyceps liangshanensis*	KUN-HKAS7723	–	–	–	–	MW168192	–	Wang et al. (2021)
*Ophiocordyceps lilacina*	YHH 2210001	–	OP782343	–	OP796856	OP796861	–	Tang et al. (2023)
*Ophiocordyceps lloydii*	OSC 151913	–	KJ878924	KJ878891	KJ878970	KJ879004	KJ878948	Quandt et al. (2014)
*Ophiocordyceps longissima*	NBRC 108989	AB968407	AB968394	AB968421	AB968585	–	–	Sanjuan et al. (2015)
*Ophiocordyceps longistipes*	KUNCC 5224	OR015962	OR082949	OR015967	OR030530	OR062224	OR113082	Fan et al. (2023)
*Ophiocordyceps longistromata*	BCC44497	MT783956	–	MT118178	MT118170	–	MT118191	Tasanathai et al. (2020)
*Ophiocordyceps macroacicularis*	NBRC 100685	–	–	AB968416	AB968574	–	AB968536	Ban et al. (2015)
*Ophiocordyceps mosingtoensis*	BCC 36921	MH754731	–	MH753685	MK284272	MK214116	MK214099	Tasanathai et al. (2019)
*Ophiocordyceps multiperitheciata*	BCC 69008	–	–	MF614657	MF614641	–	MF614682	Luangsa-ard et al. (2018)
*Ophiocordyceps myrmecophila*	CEM 1710	–	KJ878928	KJ878894	KJ878974	KJ879008	–	Quandt et al. (2014)
*Ophiocordyceps neocommunis*	HKAS 132236	PQ423674	PQ424970	PQ423693	PQ569872	PQ569886	PQ569902	Yang et al. (2025)
*Ophiocordyceps neogryllotalpae*	HAKS 131089	OR727490	OR727518	OR727504	OR735989	OR736003	OR736016	Yang et al. (2024)
*Ophiocordyceps nigrella*	EFCC 9247	JN049853	EF468963	EF468818	EF468758	EF468866	EF468920	Sung et al. (2007)
*Ophiocordyceps nooreniae*	BRIP 55363a	–	KX673811	KX673810	KX673812	–	KX673809	Crous et al. (2016)
***Ophiocordyceps northeastensis***	**HKAS 149973**	**PX692970**	**PX692983**	**PX688515**				**This study**
***Ophiocordyceps northeastensis***	**HKAS 149974**	**PX692971**	**PX692984**	**PX688516**	**PX694735**			**This study**
*Ophiocordyceps nujiangensis*	YFCC 8880	–	ON723384	ON723381	ON868820	ON868823	ON868826	Sun et al. (2022)
*Ophiocordyceps nuozhaduensis*	YHH 20168	–	ON555849	ON555927	ON567769	ON568683	–	Tang et al. (2023)
*Ophiocordyceps oecophyllae*	OECO1	–	KX713635	–	–	–	–	Araújo et al. (2018)
*Ophiocordyceps ootakii*	J13	–	KX713652	KX713600	KX713681	KX713708	–	Araújo et al. (2018)
*Ophiocordyceps ovatospora*	YHH 2206001	OP295105	OP295110	OP295113	OP313801	OP313803	OP313805	Tang et al. (2022)
*Ophiocordyceps pauciovoperitheciata*	TBRC 8106	–	–	MF614652	MF614633	–	MF614673	Luangsa-ard et al. (2018)
*Ophiocordyceps phitsanulokensis*	BCC 85328	–	–	OR805257	OR855798	OR855822	OR855843	Mongkolsamrit et al. (2024)
*Ophiocordyceps phuwiangensis*	BCC85351	MT783958	–	–	MT118174	MT118187	MT118195	Tasanathai et al. (2020)
*Ophiocordyceps ponerinarum*	HUA 186140	–	KC610789	KC610767	KC610740	KF658668	–	Sanjuan et al. (2015)
*Ophiocordyceps ponerus*	CGMCC 3.18756	KP890688	KY953152	–	KY953153	KY953154	–	Qu et al. (2018a)
*Ophiocordyceps pruinosa*	NHJ 12994	–	EU369106	EU369041	EU369024	EU369063	EU369084	Johnson et al. (2009)
*Ophiocordyceps pseudoacicularis*	TBRC 8101	–	–	MF614645	MF614629	MF614662	MF614676	Luangsa-ard et al. (2018)
*Ophiocordyceps pseudocommunis*	NHJ 12582	–	EF468975	EF468830	EF468771	–	EF468926	Tasanathai et al. (2019)
*Ophiocordyceps pseudorhizoidea*	NHJ 12529	–	EF468969	EF468824	EF468765	EF468872	EF468922	Tasanathai et al. (2019)
*Ophiocordyceps pseudovariabilis*	BCC 88308	–	–	–	OR855799	OR855823	–	Mongkolsamrit et al. (2024)
*Ophiocordyceps puluongensis*	YFCC 6442	–	MT141118	MT270528	MT270520	MT270523	MT270526	Xu et al. (2022)
*Ophiocordyceps pulvinata*	TNS F 30044	–	GU904208	–	GU904209	GU904210	–	Quandt et al. (2014)
*Ophiocordyceps purpureostromata*	TNS F18430	–	KJ878931	KJ878897	KJ878977	KJ879011	–	Quandt et al. (2014)
*Ophiocordyceps radiciformis*	BCC 93036	MZ676746	–	MZ675425	MZ707852	MZ707857	MZ707808	Tasanathai et al. (2022)
*Ophiocordyceps ratchaburiensis*	BCC 48033	–	–	OR805259	OR855802	OR855826	OR855846	Mongkolsamrit et al. (2024)
*Ophiocordyceps ravenelii*	OSC 110995	–	DQ522550	DQ518764	DQ522334	DQ522379	DQ522430	Spatafora et al. (2007)
*Ophiocordyceps robertsii*	KEW 27083	–	–	EF468826	EF468766	–	–	Sung et al. (2007)
*Ophiocordyceps rubiginosiperitheciata*	NBRC 100946	JN943341	JN941705	JN941436	AB968581	JN992439	AB968543	Ban et al. (2015)
*Ophiocordyceps rubiginosiperitheciata*	NBRC 106966	JN943344	JN941704	JN941437	AB968582	JN992438	AB968544	Ban et al. (2015)
*Ophiocordyceps salganeicola*	Mori02	–	MT741704	MT741718	MT759572	MT759579	MT759581	Araújo et al. (2021)
*Ophiocordyceps satoi*	J7	–	KX713653	KX713599	KX713683	KX713711	–	Araújo et al. (2018)
*Ophiocordyceps sinensis*	EFCC 7287	JN049854	EF468971	EF468827	EF468767	EF468874	EF468924	Sung et al. (2007)
*Ophiocordyceps sinocampes*	GZUIFR-2010MC-1	PQ765882	–	PQ766190	PQ787212	–	PQ787213	Xu et al. (2025)
*Ophiocordyceps sobolifera*	NBRC 106967	AB968409	AB968395	AB968422	AB968590	–	AB968551	Ban et al. (2015)
*Ophiocordyceps spataforae*	NHJ 12525	–	EF469125	EF469078	EF469063	EF469092	EF469111	Sung et al. (2007)
*Ophiocordyceps sphecocephala*	NBRC 101753	JN943350	JN941695	JN941446	AB968592	JN992429	AB968553	Ban et al. (2015)
*Ophiocordyceps spicatus*	MFLU 18-0164	MK863254	MK863047	MK863054	MK860192	–	–	Zha et al. (2021)
*Ophiocordyceps stylophora*	OSC 111000	JN049828	DQ522552	DQ518766	DQ522337	DQ522382	DQ522433	Spatafora et al. (2007)
*Ophiocordyceps stylophora*	OSC 110999	–	EF468982	EF468837	EF468777	EF468882	EF468931	Sung et al. (2007)
*Ophiocordyceps subtiliphialida*	YFCC 8815	–	ON555833	ON555914	ON567753	ON568673	ON568126	Tang et al. (2023)
*Ophiocordyceps superficialis*	MICH 36253	–	EF468983	–	–	EF468883	–	Sung et al. (2007)
*Ophiocordyceps taiwanensis*	TNM F0037796	PP926231	PP926233	PP926235	–	–	–	Samarakoon et al. (2024)
*Ophiocordyceps termiticola*	BCC 1770	GU723780	–	MH753677	MK284264	MK214107	MK214093	Tasanathai et al. (2019)
*Ophiocordyceps thanathonensis*	MFU 16-2909	MF850376	–	MF850377	MF872613	MF872615	–	Xiao et al. (2017)
*Ophiocordyceps tianshanensis*	MFLU 19- 1207	–	MN025409	MN025407	MK992784	–	–	Wei et al. (2020)
*Ophiocordyceps unituberculata*	YHH HU1301	–	KY923214	KY923212	KY923216	KY923218	–	Wang et al. (2018)
*Ophiocordyceps variabilis*	ARSEF 5365	–	DQ522555	DQ518769	DQ522340	DQ522386	DQ522437	Kepler et al. (2012)
*Ophiocordyceps vespulae*	GACP2017064	MN044857	–	MN044858	MN117075	–	MN107547	Long et al. (2021)
*Ophiocordyceps xifengensis*	GZUIFR Z11	OQ947874	OQ948145	OQ948160	OR014500	OR014499	–	Fei et al. (2024)
*Ophiocordyceps xuefengensis*	GZUH2012HN11	KC631800	KC631786	–	KC631791	KC631796	–	Wen et al. (2013)
*Ophiocordyceps yakusimensis*	HMAS 199604	–	KJ878938	KJ878902	–	KJ879018	KJ878953	Quandt et al. (2014)
*Paradingleyomyces lepidopterorum*	HKAS 131926	OR878363	–	OR828238	–	OR829674	OR880683	Wang et al. (2024)
*Paradingleyomyces lepidopterorum*	HKAS 131927	OR878364	–	OR828239	OR880679	OR829675	–	Wang et al. (2024)
*Paraisaria gracilioides*	HUA 186095	–	KJ917556	–	KM411994	KP212914	–	Araújo et al. (2018)
*Paraisaria gracilis*	EFCC 3101	–	EF468955	EF468810	EF468750	EF468858	EF468913	Araújo et al. (2018)
*Paraisaria heteropoda*	EFCC 10125	JN049852	EF468957	EF468812	EF468752	EF468860	EF468914	Quandt et al. (2014)
*Paraisaria heteropoda*	OSC 106404	–	AY489690	AY489722	AY489617	AY489651	–	Castlebury et al. (2004)
*Paraisaria orthopterorum*	BBC 88305	MH754742	–	MK332583	MK214080	MK214084	–	Mongkolsamrit et al. (2019)
*Paraisaria phuwiangensis*	BBH 43492	MH188541	–	MH201169	MH211355	MH211352	–	Mongkolsamrit et al. (2019)
*Paraisaria rosea*	HKAS102546	MN947222	MN943846	MN943842	MN929088	MN929081	MN929084	Wei et al. (2021)
*Paraisaria yodhathaii*	BBH43163	MH188539	–	MK332584	MH211353	MH211349	–	Mongkolsamrit et al. (2019)
*Perennicordyceps cuboidea*	NBRC 100941	JN943329	JN941725	JN941416	–	JN992459	–	Schoch et al. (2012)
*Perennicordyceps cuboideus*	CEM 1514	–	KF049609	KF049628	KF049683	–	–	Kepler et al. (2013)
*Perennicordyceps elaphomyceticola*	MFLU 21-0262	OQ172064	OQ172101	OQ172032	OQ459718	OQ459747	OQ459792	Xiao et al. (2023)
*Perennicordyceps elaphomyceticola*	NTUCC 17-021	MK840823	–	MK840812	MK839229	MK839220	MK839211	Yang et al. (2020)
*Perennicordyceps lutea*	KUMCC 3004	–	–	OQ474910	–	–	–	Xiao et al. (2023)
*Perennicordyceps paracuboidea*	NBRC 101742	JN943338	JN941710	JN941431	KF049685	JN992444	KF049669	Schoch et al. (2012)
*Perennicordyceps prolifica*	NBRC 103838	JN943339	JN941707	JN941434	–	JN992441	–	Schoch et al. (2012)
*Perennicordyceps ryogamiensis*	NBRC 101751	JN943343	JN941703	JN941438	KF049688	JN992437	–	Schoch et al. (2012)
*Perennicordyceps zongqii*	DY05421	PQ211278	–	PQ211282	PQ223679	–	PQ223677	Chen et al. (2024)
*Pleurocordyceps agarica*	YHHPA1305	KP276651	KP276655	–	KP276659	KP276663	KP276667	Wang et al. (2015a)
*Pleurocordyceps aurantiaca*	MFLUCC 17-2113	MG136916	MG136904	MG136910	MG136875	MG136866	MG136870	Xiao et al. (2019)
*Pleurocordyceps aurantiacus*	GACP 20-2306	OQ172069	OQ172098	OQ172041	OQ459715	–	OQ459789	Xiao et al. (2023)
*Pleurocordyceps clavisynnema*	GZLG 23-102	OQ968788	–	OQ968796	OQ982009	–	–	Xiao et al. (2024)
*Pleurocordyceps formosus*	MFLU 18-0162	MK863250	MK863043	MK863050	MK860188	–	–	Zha et al. (2021)
*Pleurocordyceps fusiformispora*	YFCC 07239279	PP002030	–	PP410610	PP254877	PP581807	PP581824	Liu et al. (2024)
*Pleurocordyceps heilongtanensis*	KUMCC 3008	OQ172091	OQ172111	OQ172063	OQ459731	OQ459759	OQ459805	Xiao et al. (2023)
*Pleurocordyceps kanzashianus*		AB027371	AB027325	AB027371	–	–	–	Nikoh et al. (2000)
*Pleurocordyceps lanceolata*	GACP 17-2004	OQ172076	OQ172110	OQ172046	OQ459726	OQ459754	OQ459800	Xiao et al. (2023)
*Pleurocordyceps lianzhouensis*	GIMYY9603	EU149922	KF226249	KF226250	KF226252	KF226251	–	Wang et al. (2014)
*Pleurocordyceps litangensis*	YFCC 06109293	PP410597	PP541902	PP410593	PP550103	PP697751	–	Liu et al. (2024)
*Pleurocordyceps litangensis*	YFCC 06109294	PP410598	PP541902	PP410594	PP550104	PP697752	PP550107	Liu et al. (2024)
*Pleurocordyceps litangensis*	YFCC 06109295	PP410600	PP541905	PP410596	PP550106	PP697754	–	Liu et al. (2024)
*Pleurocordyceps marginaliradians*	MFLU 17-1582	MG136920	MG136908	MG136914	MG136878	MG136869	MG271931	Xiao et al. (2019)
*Pleurocordyceps multisynnema*	GZLG 23-101	OQ968792	OQ968802	OQ968800	–	–	OQ982002	Xiao et al. (2024)
*Pleurocordyceps neoagarica*	GZLG 23-103	OQ968790	–	OQ968795	–	–	–	Xiao et al. (2024)
*Pleurocordyceps nipponica*	BCC 1881	–	KF049618	KF049636	KF049692	–	KF049674	Kepler et al. (2013)
*Pleurocordyceps nipponicus*	BCC 2325	KF049665	KF049622	KF049640	KF049696	KF049655	KF049677	Kepler et al. (2013)
*Pleurocordyceps nutansis*	MFLU 21-0275	OQ172073	OQ172119	OQ172048	OQ459739	OQ459765	OQ459811	Xiao et al. (2023)
*Pleurocordyceps nutansis*	GACP 19-1906	OQ172079	OQ172117	OQ172049	OQ459737	OQ459763	OQ459809	Xiao et al. (2023)
*Pleurocordyceps onorei*	BRA CR23902	KU898841	–	–	–	–	–	Crous et al. (2017)
*Pleurocordyceps ophiocordycipiticola*	MFLU 22-0265	OQ127364	OQ127326	OQ127397	OQ186388	OQ186435	–	Wei et al. (2022)
*Pleurocordyceps parvicapitata*	MFLU 21-0270	OQ172082	OQ172105	OQ172054	OQ459722	OQ459751	OQ459796	Xiao et al. (2023)
*Pleurocordyceps phaothaiensis*	BCC 84551	MF959731	–	MF959735	MF959739	MF959743	–	Crous et al. (2017)
*Pleurocordyceps ramosopulvinata*	SU 65	–	–	DQ118742	DQ118753	DQ127244	–	Chaverri et al. (2005)
*Pleurocordyceps ramosus*	NBRC 109983	AB925946	–	AB925982	–	–	–	Wang et al. (2020)
*Pleurocordyceps ramosus like*	NBRC 109984	MN586828	MN586819	MN586837	MN598052	MN598043	–	Wang et al. (2021)
*Pleurocordyceps sanduensis*	GZLG 23-104	OQ968786	–	OQ968798	OQ982005	–	OQ982000	Xiao et al. (2024)
***Pleurocordyceps shibingensis***	**HKAS 149965**	**PX692977**	**PX692992**	**PX688522**	**PX694731**	**PX694723**	**PX694731**	**This study**
***Pleurocordyceps shibingensis***	**HKAS 149966**	**PX692978**	**PX692993**	**PX688523**	**PX694732**	**PX694724**	**PX694732**	**This study**
*Pleurocordyceps sinensis*	CN 80-2	HQ832884	HQ832887	HQ832886	HQ832890	HQ832888	HQ832889	Wang et al. (2012)
*Pleurocordyceps sinensis*	GIMCC 3.570	JX006099	JX006097	JX006098	JX006100	JX006101	–	Zhong et al. (2016)
***Pleurocordyceps tengchongensis***	**HKAS 149971**	**PX692975**	**PX692990**	**PX688520**	**PX694741**	**PX694721**	**PX694729**	**This study**
***Pleurocordyceps tengchongensis***	**HKAS 149972**	**PX692976**	**PX692991**	**PX688521**	**PX694742**	**PX694722**	**PX694730**	**This study**
*Pleurocordyceps* sp.	NBRC 109987	AB925947	–	AB925983	–	–	–	Unpublished
*Pleurocordyceps* sp.	NBRC 109988	AB925948	–	AB925984	–	–	–	Unpublished
*Pleurocordyceps* sp.	NBRC 109990	AB925929	–	AB925968	–	–	–	Unpublished
*Pleurocordyceps* sp.	NBRC 110223	AB925930	–	–	–	–	–	Unpublished
*Pleurocordyceps* sp.	NBRC 110224	AB925931	–	AB925969	–	–	–	Unpublished
*Pleurocordyceps tomentosus*	BL4	KF049666	KF049623	KF049641	KF049697	KF049656	KF049678	Kepler et al. (2013)
*Pleurocordyceps vitellina*	KUMCC 3006	OQ172089	–	OQ172061	OQ459729	OQ459757	OQ459803	Xiao et al. (2023)
*Pleurocordyceps yunnanensis*	YHCPY 1005	KF977848	–	–	KF977850	KF977852	KF977854	Wang et al. (2015b)
*Polycephalomyces albiramus*	GACP 21-XS08	OQ172092	OQ172115	OQ172037	OQ459735	OQ459761	OQ459807	Xiao et al. (2023)
*Polycephalomyces albiramus*	GACPCC 21-XS08	OQ172093	OQ172116	OQ172038	OQ459734	OQ459762	OQ459808	Xiao et al. (2023)
***Polycephalomyces bannaensis***	**HKAS 149983**	**PX688511**	**PX692981**	**PX688513**	**PX694733**	**PX694715**		**This study**
***Polycephalomyces bannaensis***	**HKAS 149954**	**PX688512**	**PX692982**	**PX688514**	**PX694734**	**PX694716**		**This study**
***Polycephalomyces chiangraiensis***	**MFLU 26-0001**	PX692979	PX692994	PX688524	PX694745	PX694725		**This study**
***Polycephalomyces chiangraiensis***	**MFLUCC 25-0386**	**PX692980**	**PX692995**	**PX688525**	**PX694746**	**PX694726**		**This study**
*Polycephalomyces formosus*	NBRC 100686	MN586830	MN586821	MN586839	MN598054	MN598045	MN598061	Wang et al. (2020)
*Polycephalomyces formosus*	CGMCC 5.2207	MN586834	MN586825	MN586843	MN598058	MN598049	MN598065	wang et al. (2020)
*Polycephalomyces jinghongensis*	YFCC 02959283	PP274089	PP274093	PP274109	PP581803	PP697747	PP581819	Liu et al. (2024)
*Polycephalomyces jinghongensis*	YFCC 02959284	PP274090	PP274094	PP274110	PP581804	PP697748	PP581820	Liu et al. (2024)
*Polycephalomyces multiperitheciatae*	YFCC 06149287	PP274102	PP274108	PP274118	PP581802	–	PP581818	Liu et al. (2024)
*Polycephalomyces multiperitheciatae*	YFCC 06149288	PP274098	PP274104	PP274114	PP581798	PP697743	PP581815	Liu et al. (2024)
*Polycephalomyces myrmecophilus*	YFCC 09289443	PP410602	PP410608	PP410605	PP581795	PP697740	PP581812	Liu et al. (2024)
*Polycephalomyces myrmecophilus*	YFCC 09289444	PP410603	PP410609	PP410606	PP581796	PP697741	PP581813	Liu et al. (2024)
*Polycephalomyces tengchongensis*	HKAS 131923	OR878365	PP129612	OR828240	–	OR829676	OR880685	Wang et al. (2024)
*Purpureocillium atypicola*	CBS 744.73	GU980041	EF468987	EF468841	EF468786	EF468892	–	Sung et al. (2007)
*Purpureocillium jiangxiense*	JX13B01	PP555637	–	PP555646	PP658210	–	–	Chen et al. (2024)
*Purpureocillium lavendulum*	FMR 10376	–	–	FR775489	FR775516	FR775512	–	Perdomo et al. (2013)
*Purpureocillium lilacinum*	CBS 431.87	AY624188	–	EF468844	EF468791	EF468897	EF468940	Kepler et al. (2012)
*Purpureocillium roseum*	IOM 325363.1	MT560195	–	MT560197	–	–	–	Calvillo-Medina et al. (2021)
*Purpureocillium sodanum*	IBRC-M 30175	KX668542	–	–	–	–	–	Hyde et al. (2016)
*Purpureocillium takamizusanense*	NHJ_3497	–	EU369096	EU369033	EU369014	EU369053	EU369074	Johnson et al. (2009)
*Purpureocillium zongqii*	TK041	PQ211280	–	PQ211284	PQ223681	–	–	Chen et al. (2024)
*Tolypocladium album*	CBS 393.89	MH862176	–	MH873866	–	–	–	Vu et al. (2019)
*Tolypocladium amazonense*	LA100	HQ022485	KF747309	KF747129	KF747094	KF747208	–	Gazis et al. (2014)
*Tolypocladium cylindrosporum*	ARSEF 2920	MG228381	–	MH871712	MG228390	MG228384	MG228387	Vu et al. (2019)
*Tolypocladium dujiaolongae*	ZBAH632	KF696557	–	–	–	–	–	Li et al. (2018)
*Tolypocladium endophyticum*	MS337	–	KF747315	KF747136	KF747101	KF747215	–	Dong et al. (2022)
*Tolypocladium flavonigrum*	BCC66576 =MY08887	MN338090	–	MN337287	MN338495	–	–	Crous et al. (2020)
*Tolypocladium geodes*	CBS 723 70	NR_164431	–	–	–	–	–	Vu et al. (2019)
*Tolypocladium globosum*	KNUF-22-14A	LC731698	LC731700	LC731699	–	–	–	Das et al. (2023)
*Tolypocladium inflatum*	OSC 71235	JN049844	EF469124	EF469077	EF469061	EF469090	EF469108	Kepler et al. (2012)
*Tolypocladium inusitaticapitatum*	HKAS 112152	MW537735	MW537733	MW537718	MW507527	–	MW507529	Yu et al. (2021)
*Tolypocladium nubicola*	CBS 568.84	–	–	MH873478	–	–	–	Vu et al. (2019)
*Tolypocladium ophioglossoides*	NBRC 106332	JN943322	JN941732	JN941409	–	JN992466	–	Schoch et al. (2012)
*Tolypocladium paradoxum*	NBRC:100945	JN943323	JN941731	JN941410	AB968599	JN992465	AB968560	Ban et al. (2015)
*Tolypocladium pennsylvanicum*	CPC 45907	PQ498950	–	PQ498999	PQ497746	PQ497759	–	Crous et al. (2024)
*Tolypocladium phycosomatis*	TFCC 24099493	PQ864791	PQ864792	PQ864789	PV017452	PV017453	–	Li et al. (2025)
*Tolypocladium pseudoalbum*	YFCC 875	–	OP207717	OP207737	OP223151	OP223129	OP223139	Dong et al. (2022)
*Tolypocladium reniformisporum*	YFCC 1805002	–	MK984566	MK984578	MK984570	MK984585	MK984574	Dong et al. (2022)
*Tolypocladium subparadoxum*	YFCC 879	–	OP207716	OP207736	OP223150	OP223128	OP223138	Dong et al. (2022)
*Tolypocladium subtropicale*	JMS200	ON490898	–	ON495714	ON512593	ON512625	–	Soares et al. (2023)
*Tolypocladium terrae*	KNUF-23-321C	PQ773315	–	PQ773314	PQ772836	–	–	Lim et al. (2025)
*Tolypocladium trecense*	JMS111	ON490895	–	ON495712	ON512590	ON512645	–	Soares et al. (2023)
*Tolypocladium tropicale*	CBS 136897	–	–	KF747125	KF747090	KF747204	–	Gazis et al. (2014)
*Tolypocladium tundrense*	CBS:569.84	MH861781	–	MH873479	–	–	–	Bissett et al. (1983)
*Tolypocladium valdiviae*	LSB 131	OP345930	OP345933	OP345929	–	–	–	Gallardo-Pillancari et al. (2023)
*Tolypocladium yunnanense*	YFCC 877	–	OP207719	OP207739	OP223153	OP223131	–	Dong et al. (2022)
*Torrubiellomyces zombiae*	NY04434801	–	ON493543	ON493602	ON513396	ON513398	ON513402	Araújo et al. (2022)
*Torrubiellomyces zombiae*	FieldB	–	ON493544	ON493603	ON513395	–	–	Araújo et al. (2022)
*Cordyceps militaris*	OSC 93623	JN049825	AY184977	AY184966	DQ522332	DQ522377	AY545732	Kepler et al. (2012)
*Cordyceps militaris*	YFCC 6587	–	MN576762	MN576818	MN576988	MN576878	MN576932	Wang et al. (2020)

### Phylogenetic analysis

The forward and reverse reads generated in this study were assembled using BioEdit v.7.0.5.3, and the initial identification was performed by BLAST searches in GenBank. Based on the latest literature, updated sequence data were obtained from GenBank. The One-click Fungal Phylogenetic Tool (OFPT) framework (Zeng et al. 2023) was used to construct an initial comprehensive phylogenetic tree for *Ophiocordycipitaceae* and *Polycephalomycetaceae*, which was subsequently refined. Single-gene sequence alignments were conducted using the online program MAFFT v.7.110 (https://mafft.cbrc.jp/alignment/server/). TrimAl v1.2 (http://trimal.cgenomics.org) was used to remove uninformative gaps and ambiguous regions (Capella-Gutiérrez et al. 2009), and SequenceMatrix v.1.7.8 was used to concatenate the individual alignments (Vaidya et al. 2011). The final alignment was converted to NEXUS format using AliView v.1.28 (Larsson 2014). Maximum likelihood (ML) and Bayesian inference (BI) algorithms were used to perform phylogenetic analyses of the aligned sequences on the CIPRES Science Gateway portal (www.phylo.org) (Miller et al. 2010). Maximum likelihood analysis was performed using RAxML-HPC BlackBox with rapid bootstrap analysis, followed by 1,000 bootstrap replicates; the GTRGAMMA model was applied to all partitions. Bayesian analysis was used to evaluate posterior probabilities (PP) using MrBayes on XSEDE v.3.2.7a; six simultaneous Markov chains were run for 2 million generations, and trees were sampled every 200 generations (resulting in 10,000 trees). The analysis was terminated when convergence was achieved and the average standard deviation of split frequencies fell below 0.01. Phylogenetic trees were visualized using FigTree v.1.4.2 (Rambaut 2014) and edited using Adobe Illustrator CS6 (Adobe Systems Inc., USA). Maximum likelihood bootstrap values equal to or greater than 70% and PP values equal to or greater than 0.90 were shown above the nodes.

### Genealogical concordance phylogenetic species recognition analysis

To assess recombination levels among closely related species, genealogical concordance phylogenetic species recognition (GCPSR) was applied using the pairwise homoplasy index (PHI) test (Bruen et al. 2006). Relationships among closely related taxa were visualized by constructing a phylogenetic network based on a concatenated dataset of five loci (ITS, nrSSU, nrLSU, *tef*1-α, and *rpb*1) using the LogDet transformation in SplitsTree v.4 (Huson and Bryant 2006). A Φw value ≤ 0.05 was interpreted as statistically significant evidence of recombination within the dataset. This analysis focused specifically on *Polycephalomyces bannaensis* due to unresolved phylogenetic relationships. In contrast, other species included in this study did not exhibit such ambiguity and were readily distinguishable using standard phylogenetic methods; therefore, additional recombination analyses were not required.

## Results

### Phylogenetic analyses

**Figure 1.**
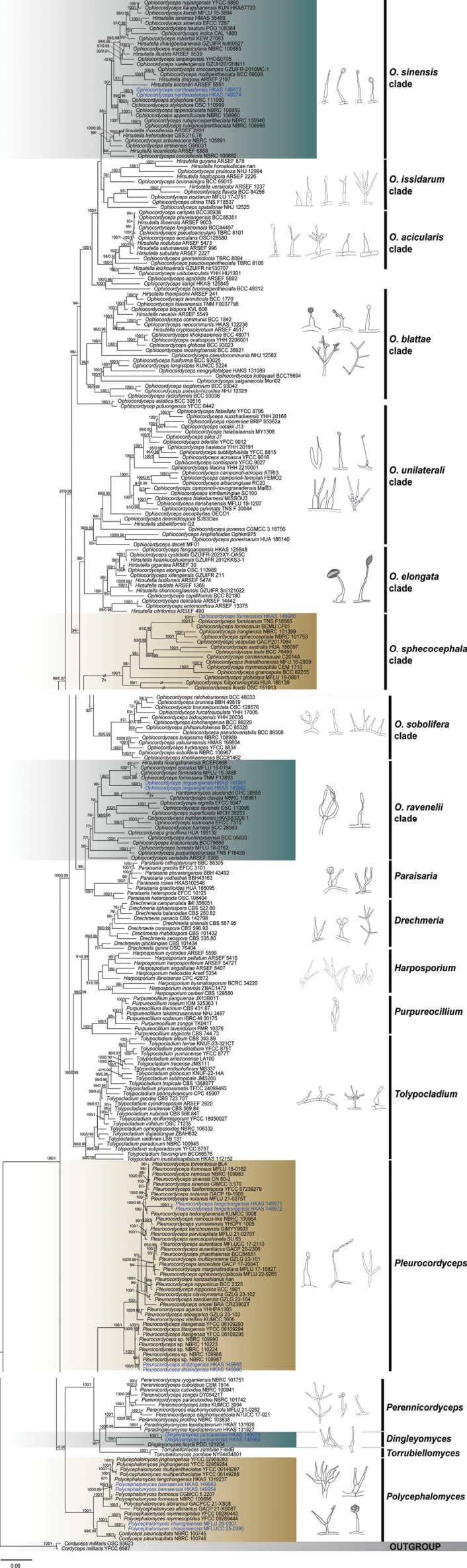
Phylogram generated from maximum likelihood analysis based on combined nrLSU, ITS, nrSSU, *tef*1-α, *rpb*1, and *rpb*2 sequence data. ML bootstrap values equal to or greater than 70% and PP values equal to or greater than 0.90 are shown above the nodes. Newly generated sequences are indicated in blue.

Phylogenetic analyses were conducted using sequence data from six loci (nrLSU, ITS, nrSSU, *tef*1-α, *rpb*1, and *rpb*2), representing 303 taxa from the families *Ophiocordycipitaceae* and *Polycephalomycetaceae*. Two strains of *Cordyceps militaris* (OSC 93623 and YFCC 6587) were used as outgroup taxa. The concatenated alignment consisted of 5,072 characters, including gaps, comprising 844 bp for nrLSU, 570 bp for ITS, 1,025 bp for nrSSU, 921 bp for *tef*1-α, 688 bp for *rpb*1, and 1,024 bp for *rpb*2. The best-scoring maximum likelihood tree, with a log-likelihood value of −128081.371, is presented in Fig. 1.

The phylogenetic analysis included eight genera of *Ophiocordycipitaceae* (*Drechmeria*, *Hantamomyces*, *Harposporium*, *Ophiocordyceps*, *Paraisaria*, *Purpureocillium*, *Tolypocladium*, and *Torrubiellomyces*), five genera of *Polycephalomycetaceae* (*Dingleyomyces*, *Paradingleyomyces*, *Perennicordyceps*, *Pleurocordyceps*, and *Polycephalomyces*), and one incertae sedis taxon (*Cordyceps pleuricapitata*) within *Hypocreales*. *Ophiocordyceps* was represented by nine distinct clades: *O. acicularis*, *O. blattae*, *O. elongata*, *O. issidarum*, *O. ravenelii*, *O. sinensis*, *O. sphecocephala*, *O. sobolifera*, and *O. unilateralis*. *Ophiocordyceps northeastensis***sp. nov**. is sister to *O. stylophora* (99% ML/0.98 PP; Fig. 1) and nests within the *O. sinensis* clade. *Ophiocordyceps jinguangensis***sp. nov**. is sister to a clade containing *O. formosana*, *O. spicatus*, and *H. huangshanensis*, with strong support (100% ML/1.00 PP; Fig. 1), and nests within the *O. ravenelii* clade. *Ophiocordyceps formicarum* is sister to a clade including *O. irangiensis*, *O. sphecocephala*, and *O. vespulae* (91% ML/0.99 PP; Fig. 1), grouping within the *O. sphecocephala* clade. Additionally, five hirsutella-like species (*Polycephalomyces bannaensis***sp. nov**., *Po. chiangraiensis***sp. nov**., *Pleurocordyceps shibingensis***sp. nov**., *Pl. tengchongensis***sp. nov**., and *Dingleyomyces yunnanensis***sp. nov**.) were confirmed as members of *Polycephalomycetaceae*. Collectively, hirsutella-like species within *Ophiocordycipitaceae* and *Polycephalomycetaceae* comprise 17 distinct clades. In contrast, the *O. sphecocephala* clade and the *Torrubiellomyces* clade, although present in the overall phylogram, do not belong to the hirsutella-like lineage as defined in this study. All clades are supported by high bootstrap values and Bayesian posterior probabilities (Fig. 1).

**Figure 2.**
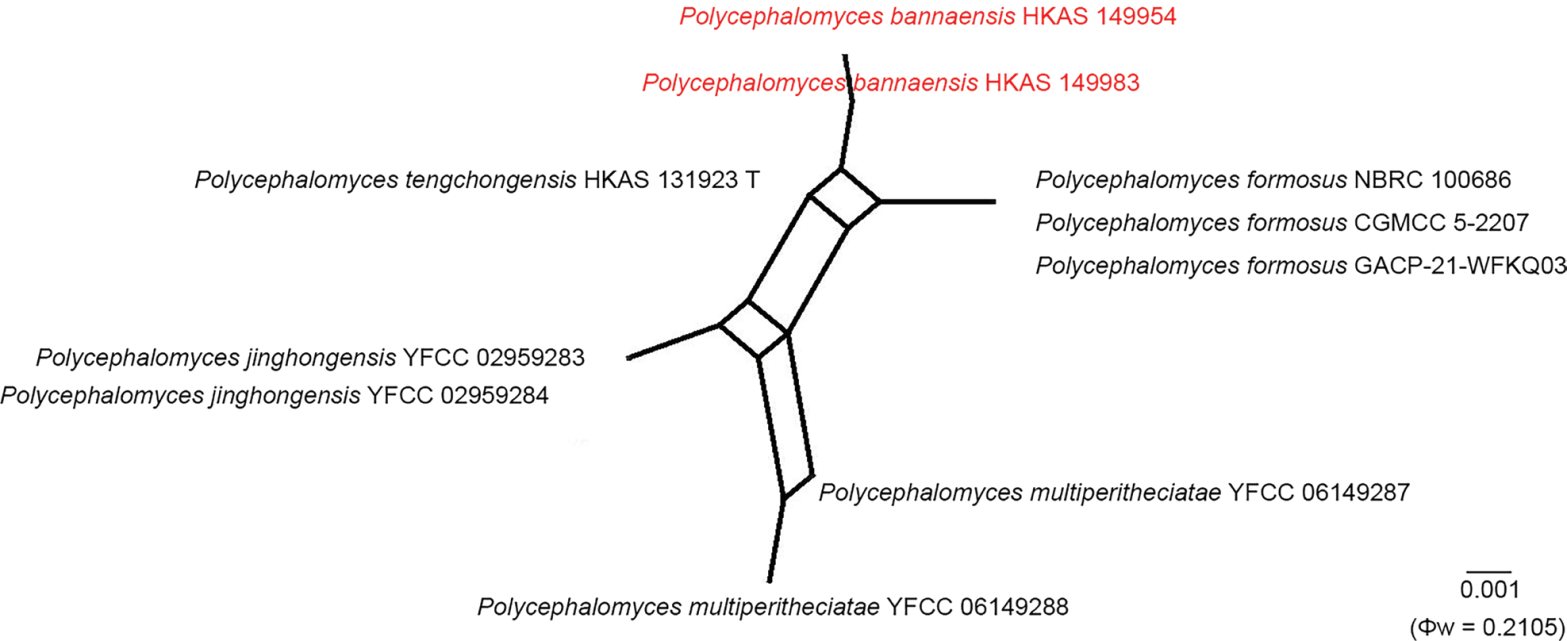
Split graphs showing results of the pairwise homoplasy index (PHI) test of the new taxa and closely related taxa using LogDet transformation and splits decomposition.

A pairwise homoplasy index (PHI) test was conducted using a five-gene dataset (ITS, nrSSU, nrLSU, *tef*1-α, and *rpb*1) to assess recombination levels among clades of *Polycephalomyces bannaensis***sp. nov**., *Po. tengchongensis*, *Po. multiperitheciatae*, *Po. jinghongensis*, and *Po. formosus*. No significant recombination events were detected among these groups (Φw > 0.05), indicating genetic isolation and supporting their recognition as distinct species (Fig. 2).

### Taxonomy

#### Ophiocordycipitaceae

Taxon classificationAnimaliaOphiocordycipitaceae

G.H. Sung, J.M. Sung, Hywel-Jones & Spatafora, 2007.

0D3DB5B5-A52A-57D7-8F70-9B9C1A950B89

Ophiocordyceps Petch, 1931.

##### Notes.

*Ophiocordyceps (Ophiocordycipitaceae)* is a genus of entomopathogenic fungi encompassing over 350 species (Hyde et al. 2024; Index Fungorum 2025). This genus was originally treated as a subgenus under *Cordyceps* by Petch (1931) until it was redefined as a distinct genus through multigene phylogenetic analyses by Sung et al. (2007). Species in this genus exhibit diagnostically dark, fibrous to wiry stromata with variable morphologies (clavate, filiform, or branched) and perithecia that are either superficially exposed or embedded within the stromatal matrix. The asci are typically cylindrical and bear prominent apical caps, while the ascospores are generally cylindrical, multiseptate, and may either disarticulate into part-spores or remain intact after discharge (Sung et al. 2007; Ban et al. 2015). Host specificity spans diverse arthropods, parasitizing larvae of *Lepidoptera*, *Coleoptera*, *Hymenoptera*, *Hemiptera*, *Diptera*, and *arachnids* (Kobayasi 1941; Mains 1958; Ban et al. 2015). Quandt et al. (2014) present a concise, thorough, phylogenetically relevant, and taxonomically accurate revision of the family *Ophiocordycipitaceae*. In this study, we introduce two new species (*O. jinguangensis* and *O. northeastensis*) based on phylogenetic and morphological analyses.

#### Ophiocordyceps jinguangensis

Taxon classificationAnimaliaOphiocordycipitaceae

X. Zhang, C.J.Y. Li, K.D. Hyde & T.C. Wen sp. nov.

6129B075-B321-57E1-9E06-45220471AFFD

Index Fungorum: IF904199

Fig. 3

##### Etymology.

Reference to the Jinguang Temple Nature Reserve, Yongping County, Dali City, the locality where the type specimen was collected.

##### Type.

CHINA • Yunnan Province, Dali City, Yongping County, Jinguang Temple Nature Reserve, 25°15'54.11"N, 99°32'13.27"E, alt. 2280 m, 25 July 2024, Cui-Jin-Yi Li, DD247162-1 (**holotype**: HKAS 149981)

##### Description.

Parasitic on a larva of ***Tenebrionoidea*** (***Coleoptera***), 37.4 × 2.8–5 mm, yellowish-brown. **Sexual morph**: ***Stroma*** arising from the abdomen and tail of the host, solitary, unbranched, yellow to brown, 10.2–16.5 × 1–2.2 mm. ***Fertile part*** up to 3.4–7.1 × 1.2–2.2 mm, cylindrical, yellow to brown, single, rough, with ostiole. ***Stipe*** clavate, light brown to brown, the color gradually becoming dark towards the apex, 7.8 × 1.0–1.2 mm. ***Perithecia*** 306–496 × 134–223 μm (x̄ = 388.4 × 175.9 µm, n = 15), immersed, ovoid to oblong-ovate. ***Asci*** 153.6–273.2 × 5.2–11.8 μm (x̄ = 234.7 × 8.4 µm, n = 20), cylindrical, hyaline, with thickened apex. ***Apical cap*** 2.5–5 × 3.5–5.6 μm (x̄ = 3.6 × 4.7 µm, n = 20), hyaline, hemispherical. ***Ascospores*** 133.4–187.6 × 1.4–2.4 µm (x̄ = 164.3 × 1.9 µm, n = 15), filiform, hyaline, easily breaking into part-spores. ***Secondary ascospores*** 6.7–9.6 × 1.6–2.7 μm (x̄ = 8.1 × 2.2 µm, n = 20), cylindrical, smooth-walled. **Asexual morph**: undetermined.

**Figure 3.**
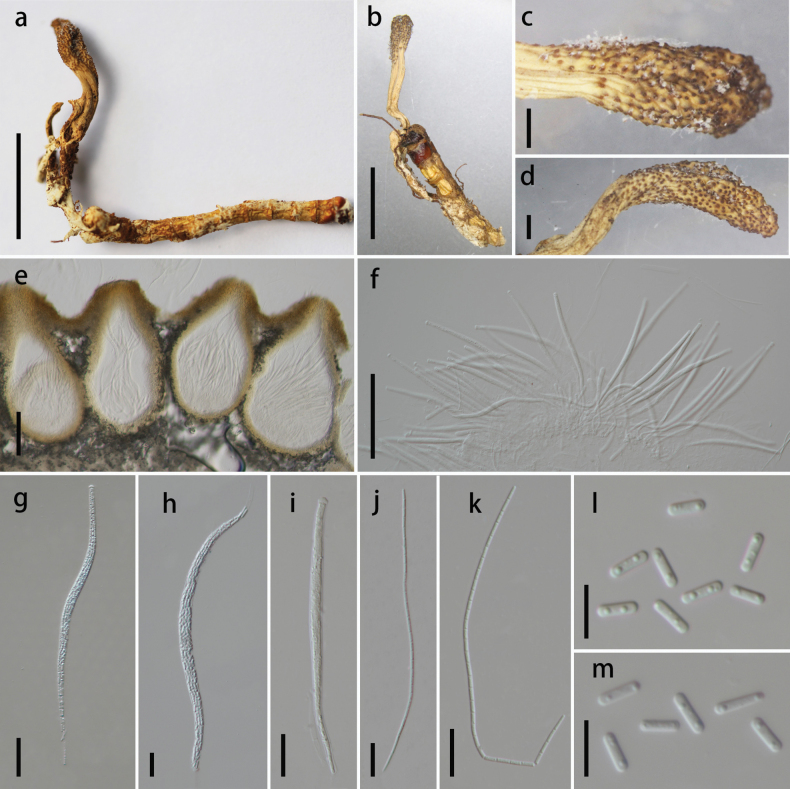
*Ophiocordyceps jinguangensis* (HKAS 149981, holotype). **A, B** stroma arising from host; **C, D** fertile part; **E** perithecia; **F** immature asci of different lengths; **G–I** asci; **J, K** ascospores; **L, M** secondary ascospores. Scale bars: 1 cm (**A**); 0.5 cm (**B**); 0.1 cm (**C, D**); 100 µm (**E, F**); 20 µm (**G–K**); 10 µm (**L, M**).

##### Habitat and distribution.

On dead larva of *Tenebrionoidea (Coleoptera)*; currently known from southwestern China.

##### Additional specimens examined.

CHINA • Yunnan Province, Dali City, Yongping County, Jinguang Temple Nature Reserve, 25°15'54.11"N, 99°32'13.27"E, alt. 2280 m, 25 July 2024, Cui-Jin-Yi Li, DD247162-2 (**paratype**: HKAS 149982).

##### Notes.

The newly described species exhibits morphological characteristics closely resembling those of *O. formosana* (Kobayasi and Shimizu 1980; Li et al. 2016) and *O. spicatus* (Zha et al. 2021), particularly in sharing features such as yellow to brown, unbranched fertile heads, cylindrical asci with the central portion slightly wider than the apical and basal regions, and filiform ascospores. Phylogenetic analysis places *O. jinguangensis* as a sister lineage to *O. formosana* and *O. spicatus*, with 100% ML/1 PP support (Fig. 1). However, *O. jinguangensis* is readily distinguished by its stroma lacking the vivid orange to yellow-orange pigmentation characteristic of *O. formosana* and *O. spicatus*. It further differs from *O. formosana* in having shorter asci (153.6–273.2 × 5.2–11.8 µm vs. 366–498 × 8–11 µm) and longer secondary ascospores (6.7–9.6 × 1.6–2.7 µm vs. 2–6 × 1–3 µm). The combined evidence from morphological characteristics and phylogenetic analyses confirms the taxonomic distinction of *O. jinguangensis*, thereby justifying its designation as a novel species.

#### Ophiocordyceps northeastensis

Taxon classificationAnimaliaOphiocordycipitaceae

X. Zhang, K.D. Hyde & T.C. Wen sp. nov.

00B0F3F1-D451-50B2-AF12-DE92CA74819C

Index Fungorum: IF904200

Fig. 4

##### Etymology.

Referring to Lushuihe Village, Fusong County, Baishan City, Jilin Province, China, the locality belongs to Northeast China, where the type specimen was collected.

##### Type.

CHINA • Jilin Province, Baishan City, Fusong County, Lushuihe Village, 42°50'33.25"N, 127°78'23.05"E, 15 November 2024, Ting-Chi Wen, DB3 (**holotype**: HKAS 149974)

##### Description.

Parasitic on larvae of ***Elateridae*** (***Coleoptera***). **Sexual morph**: undetermined. **Asexual morph**: Hyphomycetous. ***Primary synnemata*** emerging from the junction between head and tail of host, 36.6–64.5 × 0.5–0.9 mm. ***Secondary synnemata*** 4.6–25.5 mm in length, 0.7–1.5 mm in width, arising from primary synnemata, solitary, white, becoming brown with age, cylindrical, tapering gradually toward the apex. ***Phialides*** 22.3–29.0 × 1.0–4.5 µm (x̄ = 26.3 × 2.4 µm, n = 30), with swollen base and slender neck, hyaline, directly produced on superficial hyphae of secondary branches. ***Conidia*** 5.3–9.4 × 2.1–4.9 µm (x̄ = 7.6 × 3.8 µm, n = 30), unicellular, hyaline, oblong-ellipsoid to obovoid.

**Figure 4.**
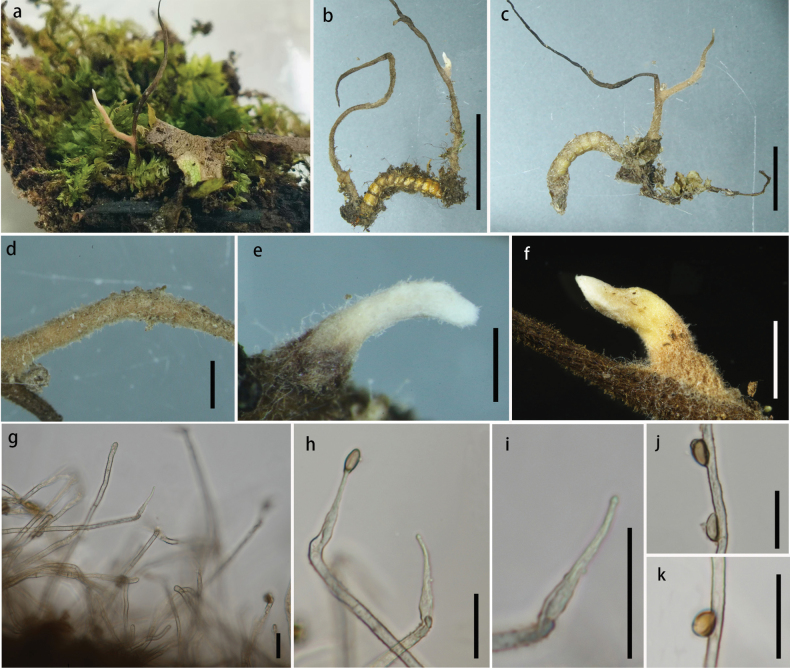
*Ophiocordyceps northeastensis* (HKAS 149974, holotype). **A** habitat of *O. northeastensis*; **B, C** synnemata arising from the host; **D–F** synnemata; **G–I** phialides; **J, K** conidia. Scale bars: 1.0 cm (**B, C**); 0.2 cm (**D**); 0.1 cm (**E, F**); 20 µm (**G–K**).

##### Habitat and distribution.

On larvae of *Elateridae (Coleoptera)* from rotten wood; currently known from northeast China.

##### Additional specimens examined.

CHINA • Jilin Province, Baishan City, Fusong County, Lushuihe Village, 42°50'33.25"N, 127°78'23.05"E, 15 November 2024, Ting-Chi Wen, DB2 (**paratype**: HKAS 149973).

##### Notes.

The newly proposed species, *O. northeastensis*, has only been observed in its asexual morph, while its sister lineage, *O. stylophora*, has been reported exclusively in its sexual morph, making it impossible to compare their morphological differences. They are proposed as distinct species based on molecular phylogenetic relationships. The new species (DB2203) differs from *O. stylophora* (OSC 111000) by 35/581 bp (6%, 9 gaps) in ITS, 3/817 bp (0.4%) in nrLSU, 389/1343 bp (28.9%, 388 gaps) in nrSSU, and 33/780 bp (4.2%) in *tef*1-α.

#### Polycephalomycetaceae

Taxon classificationAnimaliaPolycephalomycetaceae

Y.P. Xiao, Y.B. Wang, T.C. Wen, H. Yu & K.D. Hyde, 2023.

05C27D27-F091-530F-9C14-011713CA5618

Polycephalomyces Kobayasi 1941.

##### Notes.

*Polycephalomyces* is a genus established by Kobayasi (1941), with *Po. formosus* as its type species. Kepler et al. (2013) reclassified several species into *Polycephalomyces* based on molecular phylogenetic analyses, confirming its placement within *Ophiocordycipitaceae* under the “One Fungus One Name” principle. This principle unifies the taxonomies of sexual and asexual morphs (Hawksworth et al. 2011; Kepler et al. 2013; Quandt et al. 2014). Matočec et al. (2014) proposed a new genus, *Perennicordyceps*, for four *Polycephalomyces* species based on their distinct morphology, including superficial perithecia and hirsutella- or acremonium-like anamorphs. Xiao et al. (2023) transferred *Polycephalomyces* to *Polycephalomycetaceae* based on morphological and phylogenetic analyses. Species within this genus exhibit a broad host range, parasitizing insects and other fungi (e.g., *Ophiocordyceps* spp. and *Elaphomyces* spp.), highlighting their ecological versatility (Bischoff et al. 2005; Van Vooren and Audibert 2005; Ban et al. 2009; Wang et al. 2012; Matočec et al. 2014; Wang et al. 2015a, 2015b; Xiao et al. 2018; Sun et al. 2019; Wang et al. 2021; Xiao et al. 2023). In this study, we describe two new species of *Polycephalomyces*, further expanding the known diversity of this ecologically and taxonomically significant genus.

#### Polycephalomyces bannaensis

Taxon classificationAnimaliaOphiocordycipitaceae

X. Zhang, N.Y. Liu, K.D. Hyde & T.C. Wen sp. nov.

5D9CA861-AC55-54FD-959C-5687924066F0

Index Fungorum: IF904201

Fig. 5

##### Etymology.

Reference to the Mengzhe Village, Menghai County, Xishuangbanna City, the locality where the type specimen was collected.

##### Type.

CHINA • Yunnan Province, Dai autonomous prefecture of Xishuangbanna, Menghai County, Mangunxiazhai Town, 21°58'32.33"N, 100°23'5.03"E, alt. 1237 m, 10 August 2024, Nan-Yi Liu, BN24081002-1 (**holotype**: HKAS 149983).

##### Description.

Parasitic on adult of ***Coleoptera***. **Sexual morph**: undetermined. **Asexual morph**: Hyphomycetous. ***Synnemata*** 9–33 mm in length and 0.7–1.3 mm in width, occurring either scattered or clustered on the stipe, branched, white, cylindrical, with an enlarged globose fertile head at the apex. ***Fertile heads*** 0.1–0.8 mm in width, globose to subglobose, white, covered with conidial mass. ***Conidiophores*** 15–25 µm long (x̄ = 18.8 µm, n = 30), predominantly concentrated within the fertile head, monothetic, occurring either solitarily or in acropleurogenous whorls, and bear 1–4 phialides. ***Phialides*** 10–18.9 × 0.9–2.2 µm (x̄ = 13.8 × 1.5 µm, n = 30), narrowly cylindrical, hyaline, smooth-walled, tapering gradually from the middle to the apex. ***Conidia*** 2.4–3.3 × 1.5–2.1 µm (x̄ = 2.9 × 1.8 µm, n = 30), unicellular, oblong-ellipsoid to obovoid, hyaline.

**Figure 5.**
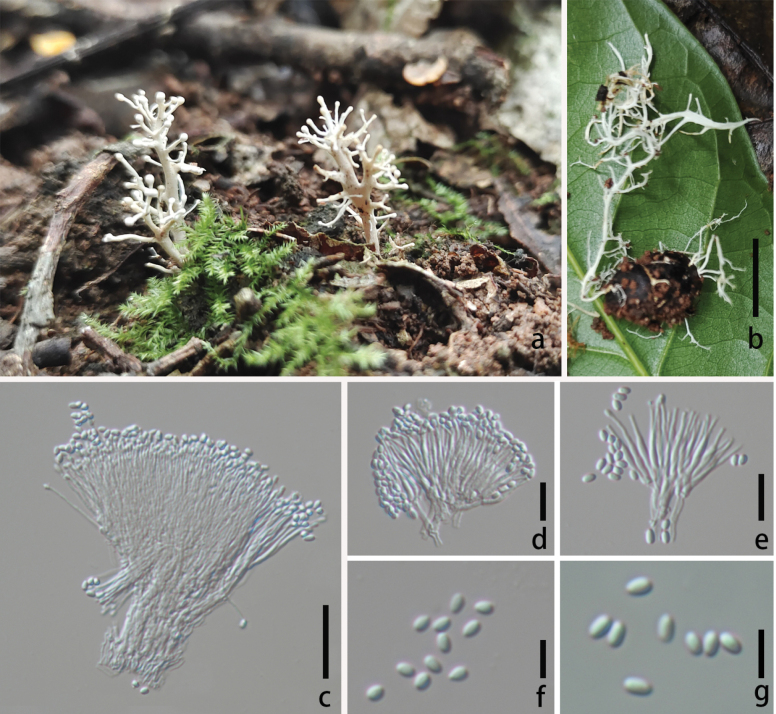
*Polycephalomyces bannaensis* (HKAS 149983, holotype). **A** habitat of *Polycephalomyces bannaensis*; **B** overview of host; **C–E** conidiophores, phialides, and conidia; **F, G** conidia. Scale bars: 1 cm (**B**); 20 µm (**C**); 10 µm (**D, E**); 5 µm (**F, G**).

##### Habitat and distribution.

On insect of *Coleoptera* currently known from southwestern China.

##### Additional specimens examined.

CHINA • Yunnan Province, Dai autonomous prefecture of Xishuangbanna, Menghai County, Mangunxiazhai Town, 21°58'32.33"N, 100°23'5.03"E, alt. 1237 m, 10 August 2024, Xing-Can Peng, BN24081002-2 (**paratype**: HKAS 149954).

##### Notes.

Phylogenetically, *Polycephalomyces bannaensis* is sister to *Po. tengchongensis* (97% ML/1 PP, Fig. 1). *Polycephalomyces bannaensis* is distinct in having longer synnemata (9–33 mm long) and oblong-ellipsoid to obovoid conidia, while *Po. tengchongensis* has shorter synnemata (18.7 mm long) and globose conidia. Additionally, *P. bannaensis* parasitizes coleopteran insects, whereas *Po. tengchongensis* infects fungi (e.g., *Perennicordyceps cf. elaphomyceticola*). The PHI test results (Fig. 2) revealed no significant recombination relationships between *Po. bannaensis* and its phylogenetically related taxa (Φw = 0.2105). Based on molecular phylogenetic analyses and morphological observations, we propose that these two species are distinct from each other and introduce our collections as a new species.

#### Polycephalomyces chiangraiensis

Taxon classificationAnimaliaOphiocordycipitaceae

X. Zhang, K.D. Hyde & T.C. Wen sp. nov.

3C1904F1-45CF-54B5-8022-5D8910129A3C

Index Fungorum: IF904830

Fig. 6

##### Etymology.

Reference to Mueang Chiang Rai District, Chiang Rai Province, the locality where the type specimen was collected.

##### Type.

THAILAND • Chiang Rai Province, Mueang Chiang Rai District, 20°02'48.30"N, 99°49'31.51"E, alt. 410 m, 28 October 2023, Xian Zhang, TX4-1 (**holotype**: MFLU 26-0001; ex-type culture: MFLUCC 25-0386).

##### Description.

Parasitic on ***Ophiocordyceps*** sp. (***Ophiocordycipitaceae***, ***Hypocreales***). **Sexual morph**: Undetermined. **Asexual morph**: Hyphomycetous. Colonies on PDA growing slowly, attaining a diameter of 1.4–1.9 cm in 21 days at 25 °C, powdery, vary in color from yellow to white, reverse yellow to brown. ***Conidiophores*** normally with 1–2 phialides. ***Phialides*** 8.2–19.8 (x̄ = 15.2 µm, n = 10) long, base 1.6–2.2 μm wide, neck 0.6–0.7 μm wide, hyaline, smooth, narrowly subulate with a neck. ***Conidia*** 2–3.2 × 0.9–1.5 µm (x̄ = 2.5 × 1.1 µm, n = 30), one-celled, hyaline, ellipsoid.

**Figure 6.**
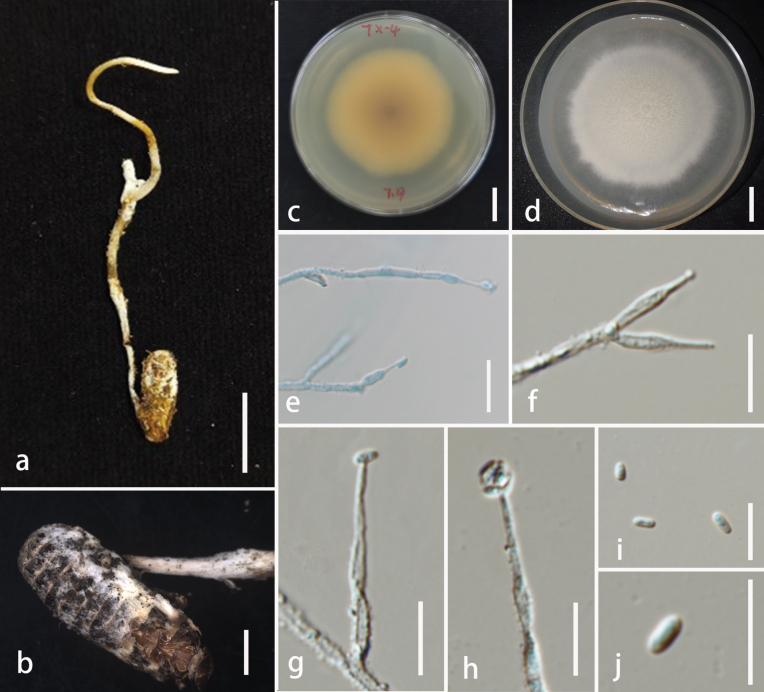
*Polycephalomyces chiangraiensis* (MFLU 26-0001, holotype). **A** overview of *Polycephalomyces chiangraiensis*; **B** overview of insect; **C, D** culture on PDA from obverse and reverse; **E–H** phialides with conidia; **I, J** conidia. Scale bars: 10 mm (**A**); 2 mm (**B**); 1 cm (**C, D**); 10 µm (**E–J**).

##### Habitat and distribution.

On *Ophiocordyceps* sp., currently known from northern Thailand.

##### Notes.

The newly proposed species, *Po. chiangraiensis*, forms a sister lineage to *Po. myrmecophilus* with 100% ML/1 PP support (Fig. 1). Morphologically, *Po. chiangraiensis* differs from *Po. myrmecophilus* by having a more pronounced difference in width between the base and apex of the phialides and ellipsoid spores. Based on these phylogenetic and morphological distinctions, *Po. chiangraiensis* is proposed as a novel species within the genus *Polycephalomyces*.

#### Pleurocordyceps

Taxon classificationAnimaliaOphiocordycipitaceae

Y.J. Yao, Y. H. Wang, S. Ban, W.J. Wang, Y. Li, K. Wang & P.M. Kirk, 2021.

9E5BB7BD-843D-51EC-B1CF-6A6823916789

##### Notes.

*Pleurocordyceps* is a genus of entomopathogenic fungi within the family *Polycephalomycetaceae*, comprising 23 species (Index Fungorum 2025). This genus was established by Wang et al. (2021) to accommodate species previously classified under *Polycephalomyces* and *Cordyceps*, with *Pleurocordyceps sinensis* (synonym: *Paecilomyces sinensis*) designated as the type species. Alongside *Polycephalomyces* and *Perennicordyceps*, it forms a well-supported monophyletic clade within *Hypocreales*. Xiao et al. (2023) further clarified its taxonomic placement by formally assigning *Pleurocordyceps* to the newly established family *Polycephalomycetaceae*, thereby stabilizing its systematic position within *Hypocreales*. The sexual morph is characterized by lateral, pulvinate stromata near the host surface, bearing perithecia close to the apex, while the asexual morph produces two distinct conidial types in culture (Wang et al. 2021). Herein, we introduce *Pl. shibingensis* and *Pl. tengchongensis* as two new species.

#### Pleurocordyceps shibingensis

Taxon classificationAnimaliaOphiocordycipitaceae

X. Zhang, K.D. Hyde & T.C. Wen sp. nov.

A254D637-A2D1-51C5-84C0-C88201D96087

Index Fungorum: IF904202

Fig. 7

##### Etymology.

Reference to Shibing County, Guizhou Province, the locality where the type specimen was collected.

##### Type.

CHINA • Guizhou Province, Shibing County, 27°09'03.55"N, 108°15'20.08"E, 22 April 2022, Xian Zhang, SB2206 (**holotype**: HKAS 149965; ex-holotype culture GACP SB2206).

##### Description.

Parasitic on larva of ***Scarabaeoidea*** (***Coleoptera***), 2.9–4 × 0.5–0.9 cm. **Sexual morph**: undetermined. **Asexual morph**: Hyphomycetous. ***Stroma*** arising from the head or abdomen of insect, 3–10 × 0.15–1.2 cm, solitary to multiple, flexuous, cylindrical to obclavate, unbranched, yellowish to off-white. Fertile part erects, subglobose, off-white, 1.2 × 0.8 cm. ***Stipe*** flexuous, yellowish, 2.3–95 × 0.15–0.25 cm, cylindrical. ***Conidiophores*** 28.1–43.7 μm (x̄ = 36.1 µm, n = 20) in length, predominantly concentrated on the fertile head, and bear 1–6 phialides. ***Phialides*** 13.3–21.9 × 1.1–1.9 μm (x̄ = 17.1 × 1.4 µm, n = 30), slender, cylindrical, hyaline, smooth-walled, arising from irregularly branched or quasi-verticillate conidiophores. ***Conidia*** 2.8–3.6 × 2.2–2.6 µm (x̄ = 3.2 × 2.5 µm, n = 30), one-celled, obovoid, hyaline, smooth-walled, guttulate, and of a single type.

***Culture characteristics***: Colonies on PDA, attaining a diameter of 23–25 mm within 14 d at 25 °C, dense, leathery, and vary in color from brown to white, thin, in the middle of the white part grew water droplets, reverse yellow with yellowish pigment. ***Conidiophores*** 26–35.8 μm long (x̄ = 30.1 µm, n = 20), concentrated, normally with 2 phialides, rarely with 6 phialides. ***Phialides*** 11.6–19.7 × 0.8–2 μm (x̄ = 15.2 × 1.4 µm, n = 30), hyaline, smooth-walled, elongated lageniform, tapering gradually from the base to the apex. ***Conidia*** 1.5–2.4 × 1–1.7 μm (x̄ = 1.8 × 1.3 µm, n = 30), one-celled, hyaline, ellipse to obovoid.

**Figure 7.**
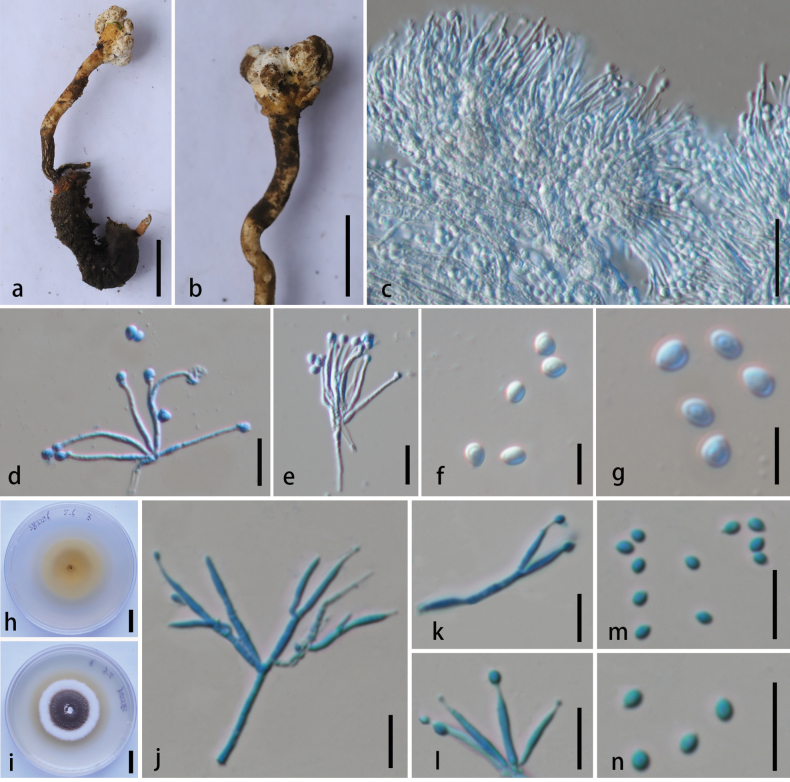
*Pleurocordyceps shibingensis* (HKAS 149965, holotype). **A** overview of host; **B** synnemata; **C, J** conidiophores; **D, E, K, L** phialides; **F, G, M, N** conidia; **H, I** culture on PDA from above and below. Scale bars: 1 cm (**A, B**); 20 µm (**C**); 10 µm (**D, E, J–N**); 5 µm (**F, G**); 1 cm (**H, I**). (**C–G** from specimen and **J–N** from culture.)

##### Habitat and distribution.

On dead larva of an insect (*Scarabaeoidea*, *Coleoptera*); currently known from southwestern China.

##### Additional specimens examined.

CHINA • Guizhou Province, Shibing County, 27°09'03.55"N, 108°15'20.08"E, 22 April 2022, Xian Zhang, SB2208 (**paratype**: HKAS 149966).

##### Notes.

Although both ITS and nrLSU sequences for four previously unpublished *Pleurocordyceps* strains (NBRC 109987, NBRC 109988, NBRC 109990, and NBRC 110224) are currently available in GenBank, Liu et al. (2024) used only the nrLSU sequences in their phylogenetic analysis, which showed that the strains clustered with *Pl. litangensis*. We reanalyzed them in this study using both loci. Our results show that *Pleurocordyceps* sp. NBRC 109987 and NBRC 109988 clustered with *Pl. shibingensis* in a well-supported clade (100% ML/0.99 PP, Fig. 1), while *Pl. litangensis* formed an independent branch (100% ML/1 PP, Fig. 1). Morphologically, *Pl. shibingensis* differs from *Pl. litangensis* by possessing whorled phialides and obovoid spores. Based on these distinct phylogenetic and morphological characteristics, *Pl. shibingensis* is proposed as a novel species within *Pleurocordyceps*.

#### Pleurocordyceps tengchongensis

Taxon classificationAnimaliaOphiocordycipitaceae

X. Zhang, K.D. Hyde & T.C. Wen sp. nov.

B060DFFE-21AF-50BC-B6E3-CBB1383CE277

Index Fungorum: IF904203

Fig. 8

##### Etymology.

Reference to Tengchong City, Yunnan Province, the locality where the type specimen was collected.

##### Type.

CHINA • Yunnan Province, Tengchong City, Houqiao Town, 25°11'30.63"N, 98°15'23.38"E, alt. 2152 m, 18 October 2024, Xian Zhang, HQ5 (**holotype**: HKAS 149971).

##### Description.

Parasitic on a larva of ***Lasiocampidae*** (***Lepidoptera***), 37.4 × 4.0–7.6 mm, yellowish-brown. **Sexual morph**: undetermined. **Asexual morph**: ***Synnemata*** 3.9–5.3 mm long, 0.3–0.6 mm wide, multiple, branched, cylindrical, clavate, capitate, stipitate, with fertile head at the apex, on the stromata of a larva of ***Lasiocampidae***. ***Conidiophores*** 20.5–25.5 μm (x̄ = 23.2 µm, n = 15), erect, arising from hyphae, bearing phialides aggregates or clusters, often additional branches occur which are equal in length form stipe structures, 1–4 phialides. ***Phialides*** 13.2–16.2 × 0.8–2.1 μm (x̄ = 14.3 × 1.3 µm, n = 30), gathered on the tip of synnema, slender, elongated lageniform, tapering gradually from the base to the apex, hyaline, smooth-walled. ***Conidia*** 2.3–3.3 × 1–1.7 µm (x̄ = 2.9 × 1.5 µm, n = 30), one-celled, hyaline, smooth-walled, oval.

**Figure 8.**
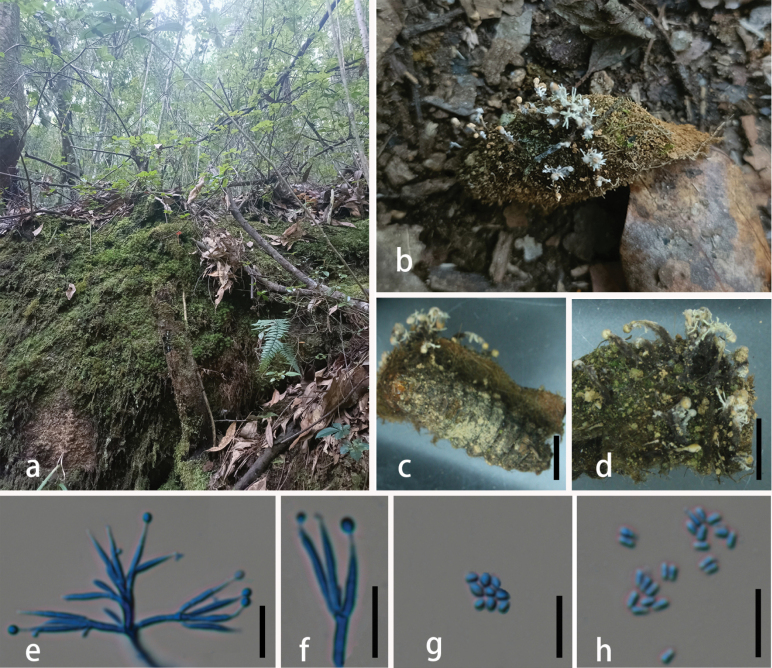
*Pleurocordyceps tengchongensis* (HKAS 149971, holotype). **A** habitat of *Pleurocordyceps tengchongensis*; **B** overview of *Pl. tengchongensis*; **C** overview of host; **D** host with synnemata; **E** conidiophores; **F** phialides; **G, H** conidia. Scale bars: 0.5 cm (**C, D**); 10 µm (**E–H**).

##### Habitat and distribution.

On dead larva of *Lasiocampidae (Lepidoptera)*; currently known from southwestern China.

##### Additional specimens examined.

CHINA • Yunnan Province, Tengchong City, Houqiao Town, 25°11'30.63"N, 98°15'23.38"E, alt. 2152 m, 18 October 2024, Xian Zhang, HQ6 (**paratype**: HKAS 149972).

##### Notes.

The newly proposed species, *Pl. tengchongensis*, forms a sister lineage to *Pl. heilongtanensis* with 100% ML/0.98 PP support (Fig. 1). Morphologically, *Pl. tengchongensis* differs from *Pl. heilongtanensis* in having hirsutella-like phialides with a rapidly tapering neck (Xiao et al. 2023). Based on these phylogenetic and morphological distinctions, *Pl. tengchongensis* is proposed as a novel species within the genus *Pleurocordyceps*.

#### Dingleyomyces

Taxon classificationAnimaliaPolycephalomycetaceae

P.R. Johnst. & D.C. Park 2023.

979BC71B-8A50-54F6-8830-4EC4FDB78824

##### Notes.

*Dingleyomyces lloydii* (synonym: *Ophionectria lloydii*) is an endemic New Zealand fungicolous fungus that parasitizes the stromata of *O. robertsii* and *O. hauturu*. Initially described invalidly as *Ophionectria cordyceps*, it was later validly published as *Ophionectria lloydii* (Mains 1958) and subsequently recombined as *Torrubiella lloydii* (Rossman 1977). However, molecular phylogenetic analyses revealed *Torrubiella* to be polyphyletic, leading to its taxonomic abandonment (Kepler et al. 2017). Johnston et al. (2023) established a new genus, *Dingleyomyces*, to accommodate this species based on integrative morphological and phylogenetic evidence. The type species is characterized by small perithecial clusters forming on reduced, non-stipitate stromata. The asexual morph is characterized by conidiophores arising from hyphae, typically bearing two phialides with swollen bases and tapering necks. Conidia are cylindric, straight, and aseptate. In this study, we introduce *Dingleyomyces yunnanensis* as a new species.

#### Dingleyomyces yunnanensis

Taxon classificationAnimaliaPolycephalomycetaceae

X. Zhang, K.D. Hyde & T.C. Wen sp. nov.

B5FE37F0-AF84-5103-84A4-1FD8CE10A283

Index Fungorum: IF904204

Fig. 9

##### Etymology.

Reference to Yunnan Province, the locality where the type specimen was collected.

##### Type.

CHINA • Yunnan Province, Tengchong City, Houqiao Town, 25°11'30.63"N, 98°15'23.38"E, alt. 2152 m, 18 October 2024, Xian Zhang, HQ4 (**holotype**: HKAS 149970).

##### Description.

Parasitic on ***Ophiocordyceps*** cf. **globiceps** (***Ophiocordycipitaceae***, ***Hypocreales***). **Sexual morph**: undetermined. **Asexual morph**: ***Synnemata*** 5.7–8.6 mm long, 0.3–0.5 mm wide, cylindrical, clavate, partial enlargement at the apex, stipitate, growing up from the head and tail of insect, multiple, white***. Stipes*** 5.1–7.9 mm long, 0.3–0.5 mm wide, cylindrical, white. ***Conidiophores*** concentrated, mono-terverticillate, and having two kinds of phialides. ***Phialides*** two types, both types observed on the same synnema. ***α-phialides*** 9.2–18.5 × 1.2–1.9 μm (x̄ = 13.4 × 1.6 μm, n = 30), hyaline, smooth, caespitose, palisade-like, crowed, gathered in the top of synnema. ***β-phialides*** 23.5–43.6 × 0.7–2.5 μm (x̄ = 32.4 × 1.7 μm, n = 30), hyaline, smooth, solitary, lanceolate, tapering into a long neck. ***Conidia*** 3–4.1 × 1.4–2.1 µm (x̄ = 3.6 × 1.7 µm, n = 30), one-celled, hyaline, smooth-walled, fusiform.

**Figure 9.**
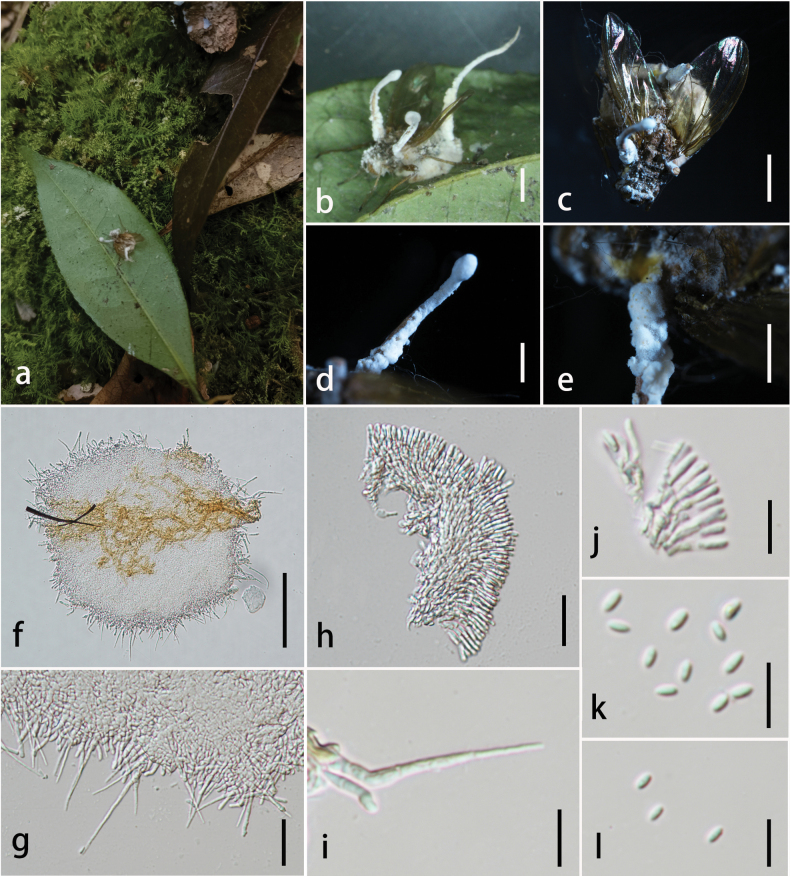
*Dingleyomyces yunnanensis* (HKAS 149970, holotype). **A** habitat of *D. yunnanensis*; **B** overview of *D. yunnanensis*; **C** overview of host; **D, E** synnemata; **F–H** conidiophores; **I** α-phialides; **J** β-phialides; **K, L** conidia. Scale bars: 0.2 cm (**B, C**); 0.1 cm (**D, E**); 100 µm (**F**); 20 µm (**G, H**); 10 µm (**I–L**).

##### Habitat and distribution.

On *Ophiocordyceps cf. globiceps* (*Ophiocordycipitaceae*, *Hypocreales*); currently known from southwestern China.

##### Additional specimens examined.

CHINA • Yunnan Province, Tengchong City, Houqiao Town, 25°11'30.63"N, 98°15'23.38"E, alt. 2152 m, 18 October 2024, Xian Zhang, HQ3 (**paratype**: HKAS 149969).

##### Notes.

The newly proposed species, *D. yunnanensis*, is a previously undescribed hirsutella-like species that forms a sister lineage to *D. lloydii* (the only currently recognized species in *Dingleyomyces*) with strong statistical support (100% ML/1 PP, Fig. 1). Morphologically, *D. yunnanensis* differs from *D. lloydii* in phialide shape and conidial morphology: *D. lloydii* has pyriform phialides and cylindrical conidia, whereas *D. yunnanensis* possesses palisade-like to lanceolate phialides and fusiform conidia. Critically, this study provides the first documentation of the asexual morph in *Dingleyomyces*. Based on these phylogenetic and morphological distinctions, *D. yunnanensis* is proposed as a novel species within *Dingleyomyces*.

## Discussion

Hirsutella-like morphologies are not only found in *Ophiocordyceps* but also occur in other genera of *Ophiocordycipitaceae*, *Polycephalomycetaceae*, and *Clavicipitaceae*, complicating the morphological delimitation of *Hirsutella*. This study re-evaluates clades bearing hirsutella-like anamorphs within *Ophiocordycipitaceae* and *Polycephalomycetaceae* by: (1) mapping the morphological diversity of hirsutella-like anamorphs onto phylogenetic trees, and (2) analyzing their host associations and ecological diversification patterns (Fig. 1).

Building upon this phylogenetic framework, we provide a detailed morphological re-examination of 17 clades possessing hirsutella-like anamorphs. The following sections describe key characteristics for each clade, such as phialide morphology, conidial features, and host affiliations. In addition, the Discussion integrates observations from the newly described species and evaluates their significance within their respective clades, including their morphological conformity or deviation, host associations, and phylogenetic placement. These reflections provide insights into patterns of character evolution, clade stability, and the evolutionary relevance of hirsutella-like morphologies, thereby clarifying taxonomic boundaries and evolutionary relationships among these taxa.

### *Ophiocordyceps sinensis* clade

The *O. sinensis* clade contains the medically significant *Ophiocordyceps sinensis* (Sung et al. 2007) and its asexual morph, *Hirsutella sinensis* (Liu et al. 1989). Phialides in this clade feature cylindrical, slender, or subulate bases that taper gradually or suddenly into distinct necks. Several species, including *H. illustris*, *H. strigosa*, *H. rhossiliensis*, *O. sinocampes*, and *O. nujiangensis*, display distinctive warty protrusions on their phialides. Notably, the newly described species *O. northeastensis* also exhibits conspicuous warty ornamentation on the phialides, further supporting its placement within the *O. sinensis* clade. Additionally, multiple species produce spores embedded in mucous sheaths, such as *H. sinensis*, *H. illustris*, *H. strigosa*, *H. rhossiliensis*, *O. liangshanensis*, and *O. multiperitheciata*. This clade exhibits remarkably broad host specificity (Simmons et al. 2015), parasitizing diverse organisms ranging from nematodes and mites to insects belonging to *Hemiptera*, *Coleoptera*, and *Lepidoptera*. From an evolutionary perspective, the recurrence of warty phialide ornamentation and mucous-sheathed conidia across multiple lineages within the *O. sinensis* clade suggests that these characters are evolutionarily conserved and may represent stable diagnostic traits. The broad host spectrum observed in this clade, in contrast, indicates that host shifts and ecological diversification have occurred without substantial modification of key asexual morphological features.

### *Ophiocordyceps issidarum* clade

The *O. issidarum* clade, originally named *Hirsutella guyana* by Simmons et al. (2015), consists of three species (*H. guyana*, *H. haptospora*, and *H. versicolor*). The phialides in this clade are characterized by a cylindrical base that abruptly narrows into a thin neck. Some species, such as *H. guyana*, *O. flavida*, and *O. brunneinigra*, exhibit branched phialides. Xie et al. (2025) later assigned this clade the name *O. issidarum*, as its sexual forms produce superficial perithecia along with filamentous, multiseptate ascospores that remain intact at maturity (Hyde et al. 2017; Luangsa-ard et al. 2018). Most members of this clade parasitize hosts in the order *Hemiptera*. However, *H. haptospora* infects insects in the order *Diptera*, while *H. kirchneri* parasitizes insects in the order *Trombidiformes*.

### *Ophiocordyceps acicularis* clade

The *O. acicularis* clade, originally described as *Hirsutella nodulosa* by Simmons et al. (2015), exhibits monophialidic conidiogenous cells, occasionally polyphialidic, arising laterally or terminally from hyphae. These conidiogenous cells are hyaline, smooth-walled, and taper gradually or abruptly into a slender, short neck. The phialide bases on specimens are comparatively shorter than those in cultured specimens, with the necks exhibiting helical curvature and occasionally bearing verrucose ornamentation (Tasanathai et al. 2020). In contrast, the phialides of *O. unituberculata* are distinguished by a distinctive apical spherical protrusion (Wang et al. 2018). This clade comprises many cryptic species that parasitize lepidopteran larvae, except for *H. leigongshanensis* (Tasanathai et al. 2020), which infects coleopteran larvae.

### *Ophiocordyceps blattae* clade

The *O. blattae* clade, described as *Hirsutella thompsonii* by Simmons et al. (2015), is characterized by a more swollen phialide base and a shorter neck compared to other hirsutella-like clades. Its conidia range from spherical to fusiform and exhibit either a warty surface or a mucous sheath. Members of this clade predominantly parasitize cockroaches and termites (*Blattodea*). While *O. communis* in this clade exhibits an asexual morph with intermediate characteristics between *Hymenostilbe* and *Hirsutella* (Sung et al. 2007), its close relative *O. neocommunis* was recently reported to show morphological similarity, with the closest genetic distance being classified as a hirsutella-like asexual form (Yang et al. 2025).

### *Ophiocordyceps unilateralis* clade

The *O. unilateralis* clade represents one of the most prevalent groups of ant-associated entomopathogenic fungi in tropical forests globally (Evans et al. 2011). This clade comprises three morphologically and ecologically distinct clades: the *O. unilateralis* core clade, the *O. kniphofioides* subclade, and the *O. oecophyllae* subclade (Araújo et al. 2018). The *O. kniphofioides* subclade is characterized by orange ascomata developing on thorax-emerging stromata with 360° fertile coverage, typically found on hosts in Amazonian moss carpets. In contrast, the *O. unilateralis* core clade forms dorsally pronotal stromata bearing laterally attached brown to black ascomata, consistent with its “unilateralis” epithet. *Ophiocordyceps oecophyllae* differs in producing only an asexual morph, with phialides developing directly on the host, particularly from joint regions. Molecular data further confirm that this subclade is well supported (Araújo et al. 2018). Phialides across the *O. unilateralis* clade exhibit distinct hirsutella-like morphotypes (Evans et al. 2011), characterized by swollen bases that taper either gradually or abruptly into elongated necks, longer than those observed in other clades. While some species display only a single phialide type, others produce polymorphic phialides.

### *Ophiocordyceps elongata* clade

Species in this clade are associated with diverse lepidopteran and hemipteran insects and are characterized by richly branched stromata, subulate phialides, and conidia enveloped by a mucous sheath (Mongkolsamrit et al. 2024).

### *Ophiocordyceps sobolifera* clade

This clade includes the *O. sobolifera* core clade and the *O. brunneipunctata* complex subclade, comprising pathogens associated with cicada nymphs as well as coleopteran larvae (Mongkolsamrit et al. 2024). Members of the *O. sobolifera* clade typically produce stromata ranging from reddish-brown to pale yellow, cylindrical ascomata, and immersed perithecia. The asexual forms in this clade feature phialides of variable morphology—some with a spherical base that abruptly narrows into a long filamentous neck (and may branch), while others lack such an elongated neck. The conidia are subglobose to fusoid in shape (Zou et al. 2022; Mongkolsamrit et al. 2024).

### *Ophiocordyceps ravenelii* clade

This clade comprises taxa that are pathogenic to *Coleoptera* and is characterized by yellow, orange, or brown stromata, with immersed perithecia located at apical or lateral fertile regions, and filamentous, multiseptate ascospores that disarticulate into cylindrical secondary ascospores upon maturation (Wang et al. 2015c; Mongkolsamrit et al. 2024). The asexual morph is characterized by phialides occurring singly, in opposed pairs, or in verticils, with subtly inflated bases tapering distally into slender necks (Li et al. 2005). Mucous sheaths are commonly absent in species of this clade (Wang et al. 2015c). Although the asexual morph of *O. jinguangensis* was not observed in the present study, its association with coleopteran hosts and the diagnostic features of its sexual morph are congruent with the morphological circumscription of this clade. Together with its well-supported placement in the phylogenetic analyses, these lines of evidence collectively support the inclusion of *O. jinguangensis* within this lineage.

### *Paraisaria* clade

The *Paraisaria* clade has a broad host range (Mongkolsamrit et al. 2019), infecting hosts across *Hemiptera*, *Coleoptera*, *Diptera*, *Lepidoptera*, *Orthoptera*, *Blattodea*, and *Hymenoptera*. Mongkolsamrit et al. (2019) supported its classification as a valid genus within *Ophiocordycipitaceae* due to its unique morphological features. *Paraisaria* is distinctive in producing spherical to subspherical fertile heads. Its asexual stage is linked with hirsutella-like anamorphs. The synnemata comprise verticillately branched conidiophores bearing phialides with swollen bases and thin, tapering necks. Some phialides are borne singly on vegetative hyphae. Conidia are cylindrical to fusiform and aggregated in slimy heads.

### *Drechmeria* clade

The *Drechmeria* clade is known as endoparasites of nematodes and lepidopteran larvae (David and Jeffrey 1999; Spatafora et al. 2015). However, *Drechmeria panacis* was isolated as an endophyte from *Panax notoginseng* (Yu et al. 2018), suggesting broader host-range plasticity and functional diversity within the genus. Morphologically, this clade exhibits hirsutella-like morphology characterized by conidiophores bearing verticillate or solitary phialides. The phialides display a distinct swelling near the base that tapers toward the neck. Conidial morphology is variable, ranging from balanoid, cylindrical, and subglobose to irregular shapes (Zare and Gams 2001).

### *Harposporium* clade

The *Harposporium* clade predominantly parasitizes invertebrate nematodes, with a minority of isolates derived from coleopteran insects (Kuo et al. 2008; Chen et al. 2025). This fungal group displays two discrete asexual morphologies: (1) spherical to subglobose conidiogenous cells generating arcuate conidia, frequently accompanied by accessory conidia, arthroconidia, and chlamydospores (Evans and Whitehead 2005); and (2) a hirsutella-like morphology characterized by phialides with swollen bases that taper either gradually or abruptly into well-defined necks, bearing elliptical conidia at their apices—as observed in *H. peltatum*, *H. cerberi*, and *H. anguillulae* (Hodge 1997; Evans and Whitehead 2005).

### *Purpureocillium* clade

The *Purpureocillium* clade exhibits a broad ecological distribution in terrestrial and marine environments, having been isolated from diverse insect hosts, nematodes, crop rhizospheres, soil, and even a corneal ulcer patient (Giné and Sorribas 2017; Liu et al. 2019; Calvillo-Medina et al. 2021; Yang et al. 2024; Chen et al. 2024). This clade displays two distinct asexual morphologies: (1) an acremonium-like anamorph characterized by subglobose, ellipsoidal, or cylindrical conidia aggregated in slimy heads (Luangsa-ard et al. 2011); and (2) a hirsutella-like morph featuring septate conidiophores with warty protuberances, bearing verticillate phialides that produce dry chains of unicellular, subglobose conidia with apiculate bases or limoniform structures (Chen et al. 2024).

### *Tolypocladium* clade

*Tolypocladium* contains saprotrophic soil dwellers, plant endophytes, and pathogens targeting insects (e.g., mosquito larvae, fireflies, beetles, cicada nymphs, and bat moth larvae), nematodes, and rotifers, as well as parasites of truffle-like fungi (Yu et al. 2021; Dong et al. 2022). This clade exhibits hirsutella-like asexual morphs, with phialides forming singly or in whorls of 2–6. The phialides display swollen bases and narrowly tapering, frequently bent necks. Conidia are produced singly or aggregated in slimy heads (Gams 1971; Wang et al. 2022).

### *Dingleyomyces* clade

The *Dingleyomyces* clade currently contains two species (*D. lloydii* and *D. yunnanensis*). The type species heavily parasitizes *Ophiocordyceps hauturu* and *O. robertsii* stromata (Johnston et al. 2023). It represents the first lineage in the family exhibiting a torrubiella-like macromorphology while possessing a hirsutella-like anamorph. The conidiogenous cells taper toward narrow apices and arise from repeatedly branched conidiophores, which often feature swollen cells. The conidia are cylindrical, straight, aseptate, and have rounded ends. The newly described species *D. yunnanensis* expands the known host range of the clade to a different species of *Ophiocordyceps*, indicating that host specialization within *Dingleyomyces* may not be restricted to a single host lineage. Moreover, *D. yunnanensis* differs from *D. lloydii* in its asexual morphology, in which the hirsutella-like phialides are consistently solitary rather than arising from branched conidiophores. These differences in host association and phialide arrangement are consistent with its recognition as a distinct species and provide additional morphological characters for delimiting taxa within the *Dingleyomyces* clade, in agreement with the phylogenetic results.

### *Perennicordyceps* clade

*Perennicordyceps* comprises insect-parasitic taxa (parasitizing beetle larvae and cicada larvae) and fungicolous species (parasitizing *Cordyceps* spp., *Ophiocordyceps* spp., and *Elaphomyces* spp.). This clade is characterized by acremonium- and hirsutella-like asexual morphs, along with superficial perithecia. Phialides arise from irregularly branched or subverticillate conidiophores. The acremonium-like morph produces conidia (up to 10) enveloped in a mucilaginous sheath, whereas the hirsutella-like morph bears solitary conidia. Conidial morphology varies from globose to ellipsoid or limoniform to fusiform (Matočec et al. 2014; Wang et al. 2020; Xiao et al. 2023).

### *Pleurocordyceps* clade

The *Pleurocordyceps* clade parasitizes hosts across six insect orders (*Coleoptera*, *Hemiptera*, *Homoptera*, *Hymenoptera*, *Lepidoptera*, and *Orthoptera*) (Xiao et al. 2023, 2024), with additional fungicolous species infecting *Ophiocordyceps* and *Perennicordyceps* (Wang et al. 2015b; Xiao et al. 2023, 2024). The *Pleurocordyceps* clade produces acremonium- or hirsutella-like phialides and two types of conidia. However, sometimes only one type is observed in fresh specimens. These phialides branch verticillately, producing hyaline, smooth-walled conidia. Two types of conidia are usually present in culture: the α-conidia are globose to subglobose or ellipsoidal, forming masses on the fertile head, while the β-conidia are fusiform, arising singly or in chains along the stipe and mycelial surface (Xiao et al. 2023). The two newly described species, *Pleurocordyceps shibingensis* and *Pl. tengchongensis*, conform well to the established morphological circumscription and host associations of the *Pleurocordyceps* clade. Although they do not exhibit obvious morphological novelties or host-range expansions, their well-supported phylogenetic placement, together with concordant morphological characters, provides additional evidence for the stability and diagnosability of this clade. Moreover, the discovery of these species from geographically distinct regions further contributes to documenting the diversity and distribution of *Pleurocordyceps*, helping to refine species boundaries and the evolutionary framework within the clade. From an evolutionary perspective, the placement of these species supports a pattern of morphological conservatism within *Pleurocordyceps*, in which species-level diversification is reflected primarily in phylogenetic differentiation rather than conspicuous morphological or ecological shifts.

### *Polycephalomyces* clade

Species of the *Polycephalomyces* clade parasitize insects from various orders (*Orthoptera*, *Psocoptera*, *Coleoptera*, *Hymenoptera*, *Lepidoptera*, and *Hemiptera*), as well as *Ophiocordyceps* spp. and myxomycetes (Xiao et al. 2023). Its asexual morph produces synnemata on the host surface or in culture, with branched or unbranched conidiophores that are divergent. Members of the *Polycephalomyces* clade produce hirsutella-like phialides (1–4 per conidiophore) that arise terminally or intercalarily, typically featuring a narrow cylindrical base that tapers into a long neck—except in *Po. ditmarii*, where the phialides remain narrowly cylindrical (although further molecular data are needed to confirm its status). Conidia are monomorphic, oblong to cylindrical, hyaline, and smooth-walled (Xiao et al. 2023). The newly described species, *Polycephalomyces bannaensis* and *Po. chiangraiensis*, exhibit morphological characters and host associations that are highly consistent with those of previously recognized members of the clade. Although no conspicuous morphological novelties were observed, their distinct and well-supported phylogenetic positions indicate species-level divergence with limited morphological differentiation. This pattern suggests a degree of evolutionary conservatism in key morphological traits within *Polycephalomyces*, while diversification appears to be more readily captured at the molecular level. The recognition of these species from different geographic regions further contributes to refining the evolutionary framework and species delimitation within the clade.

## Conclusion

This study significantly advances our understanding of hypocrealean entomopathogenic fungi by describing seven new hirsutella-like species through multilocus phylogenetic and morphological analyses and by providing the first comprehensive overview of this morphotype across 19 clades in *Ophiocordycipitaceae* and *Polycephalomycetaceae*. Our findings clarify the morphological characteristics of hirsutella-like species in these families, documenting both conserved and divergent phialide traits among major phylogenetic lineages. The pronounced morphological diversity revealed across unrelated clades highlights the inherent limitations of relying solely on anamorphic characters for taxonomic inference. Consequently, this study underscores the importance of an integrative taxonomic framework that combines multilocus phylogeny with morphological evidence to resolve persistent taxonomic ambiguities. By mapping phialide morphologies alongside the phylogenetic tree, this work provides a refined context for future studies and emphasizes the need for continued phylogenetic sampling to further elucidate the diversity and ecological roles of these significant fungal groups.

## Supplementary Material

XML Treatment for Ophiocordycipitaceae

XML Treatment for Ophiocordyceps jinguangensis

XML Treatment for Ophiocordyceps northeastensis

XML Treatment for Polycephalomycetaceae

XML Treatment for Polycephalomyces bannaensis

XML Treatment for Polycephalomyces chiangraiensis

XML Treatment for Pleurocordyceps

XML Treatment for Pleurocordyceps shibingensis

XML Treatment for Pleurocordyceps tengchongensis

XML Treatment for Dingleyomyces

XML Treatment for Dingleyomyces yunnanensis

## Additional information

### Use of AI

No use of AI was reported.

### Adherence to national and international regulations

All the fungal strains used in this study have been legally obtained, respecting the Convention on Biological Diversity (Rio Convention).

### Author ORCIDs

Xian Zhang https://orcid.org/0009-0008-0919-4303

Xing-Can Peng https://orcid.org/0000-0002-7271-7639

De-Ping Wei https://orcid.org/0000-0002-6576-2239

Yi Wang https://orcid.org/0009-0006-5412-7893

Chada Norphanphoun https://orcid.org/0000-0002-5756-7206

Sinang Hongsanan https://orcid.org/0000-0003-0550-3152

Ning Xie https://orcid.org/0000-0002-5866-8535

Zhong-Liang Liu https://orcid.org/0009-0007-9519-1418

Ausana Mapook https://orcid.org/0000-0001-7929-2429

Ji-Chuan Kang https://orcid.org/0000-0002-6294-5793

Natsaran Saichana https://orcid.org/0000-0002-4357-7980

Kevin D. Hyde https://orcid.org/0000-0002-2191-0762

Ting-Chi Wen https://orcid.org/0000-0003-1744-5869

### Data availability

All tree alignments generated and/or analyzed during the current study were deposited in Figshare (https://doi.org/10.6084/m9.figshare.30061459).

## Supplementary materials

Supplementary material 1 Additional information Data type fas https://binary.pensoft.net/file/1542186This dataset is made available under the Open Database License (http://opendatacommons.org/licenses/odbl/1.0/). The Open Database License (ODbL) is a license agreement intended to allow users to freely share, modify, and use this Dataset while maintaining this same freedom for others, provided that the original source and author(s) are credited. Xian Zhang, Xing-Can Peng, De-Ping Wei, Yi Wang, Chada Norphanphoun, Sinang Hongsanan, Ning Xie, Zhong-Liang Liu, Ausana Mapook, Ji-Chuan Kang, Natsaran Saichana, Kevin D. Hyde, Ting-Chi Wen
